# Proteomic signatures of retinal pigment epithelium-derived exosomes in myopic and non-myopic tree shrew eyes

**DOI:** 10.3389/fmed.2025.1523211

**Published:** 2025-04-22

**Authors:** Nilda C. Sanchez, Jose Luis Roig-Lopez, James A. Mobley, Safal Khanal

**Affiliations:** ^1^School of Optometry, Department of Optometry and Vision Science, University of Alabama at Birmingham, Birmingham, AL, United States; ^2^Heersink School of Medicine, Department of Anesthesiology and Perioperative Medicine, University of Alabama at Birmingham, Birmingham, AL, United States; ^3^Heersink School of Medicine, O'Neal CCC Mass Spectrometry and Proteomics Shared Resource, University of Alabama at Birmingham, Birmingham, AL, United States

**Keywords:** myopia, exosomes, retinal pigment epithelium, tree shrews, emmetropization

## Abstract

**Purpose:**

The retinal pigment epithelium (RPE) transmits growth signals from the neural retina to the choroid in the emmetropization pathway, but the underlying molecular mechanisms remain poorly understood. Here, we compared the proteomic profiles of RPE-derived exosomes between myopic and non-myopic eyes of tree shrews, dichromatic mammals closely related to primates.

**Methods:**

Four myopic (159–210 days of visual experience, DVE) and seven non-myopic eyes (156–210 DVE) of tree shrews were included. Non-cycloplegic refractive error was measured with Nidek autorefractor, and axial ocular component dimensions were recorded with LenStar. Tissue was collected, yielding RPE-lined eyecups, which were subsequently incubated in L-15 culture media for 2 h. The RPE-derived exosomes were then enriched and purified from the incubation media by double ultracentrifugation and characterized by imaging and molecular methods. Exosomal proteins were identified and quantified with mass spectrometry, examined using GO and KEGG analyses, and compared between myopic and non-myopic samples.

**Results:**

Out of 506 RPE exosomal proteins identified, 48 and 41 were unique to the myopic and non-myopic samples, respectively. There were 286 differentially expressed proteins in the myopic samples, including 79 upregulated and 70 downregulated. The top three upregulated proteins were Histone H4 (Fold Change, FC = 3.04, *p* = 0.09), PTB 1 (FC = 2.59, *p* = 0.08) and Histone H3.1 (FC = 2.59, *p* = 0.13), while the top three downregulated proteins were RPS5 (FC = −2.41, p=0.004), ACOT7 (FC=-2.15, *p* = 0.04) and CRYBB2 (FC = −2.14, *p* = 0.05). Other differentially expressed proteins included LUM, VCL, SEPTIN11, GPX3, SPTBN1, SEPTIN7, RPL10A, KCTD12, FGG, and FMOD. Proteomic analysis revealed a low abundance of ATP6V1B2 and crystallin beta B2, and a significant depletion of the crystallin protein family (crystallin A2, A3, and B3 subunits) in the myopic samples. The enrichment analyses showed extracellular matrix, cytoskeletal dynamic, and cell-matrix adhesion as the primary components associated with the RPE exosomal proteins in myopic eyes.

**Conclusion:**

Using standard molecular and imaging techniques, this study provides the first demonstration of the *ex-vivo* RPE exosome biogenesis from tree shrew eyes. The results showed distinct differential expressions of the RPE exosomal proteins between the myopic and non-myopic eyes, with several proteins unique to each group. Future targeted proteomic studies of identified candidate exosomal protein signatures could elucidate the molecular mechanism of RPE exosome-mediated growth signal transmission in the emmetropization pathway.

## Introduction

In postnatally developing eyes, a visually guided emmetropization mechanism uses visual cues to control the rate of axial eye growth to achieve and maintain a good focus on the retina (emmetropia). Experimental alterations of visual cues, for example, by imposing defocus (1, 2) or changing the spectral composition of light (3–6), produce a compensatory modulation of eye growth, causing the eye to deviate from emmetropia. These vision-dependent changes in eye growth can occur in a regionally selective manner (7, 8) and without the need for accommodation (9, 10) or central connections to the brain (11), suggesting that the emmetropization mechanism is local to the eye and operates along the retina-choroid-sclera pathway, whereby the neurosensory retina [likely amacrine cells (12, 13)] produces a cascade of growth stimulatory (GO) or inhibitory (STOP) signals that trigger changes in choroidal thickness and scleral remodeling to control eye size and refractive state (14, 15). In the past few decades, certain environmental factors, likely related to the modern world, have led to a failure in emmetropization in an increasingly large number of individuals, causing a rapid rise in myopia prevalence worldwide (16). The mechanistic basis of this failure is not fully understood due largely to an incomplete understanding of the molecular mechanisms involved in the early retinal growth signaling pathway of emmetropization (17).

There is growing evidence that the retinal pigment epithelium (RPE) plays a critical role in the emmetropization mechanism (18). The anatomical location of the RPE—with the neurosensory retina on the apical side and the choroid on the basal side—allows it to serve as a conduit for growth signals between the retina and the choroid (15, 19, 20). During experimental manipulations of image focus, the RPE secretes growth-regulatory factors and shows bi-directional changes in gene expressions (18). For instance, eye growth-promoting stimuli cause downregulation of the BMP2 (21–25), while growth-inhibiting stimuli cause upregulation (21, 23, 25). These results provide compelling evidence for RPE-mediated control of eye growth, although how the RPE transmits signaling information related to eye growth remains unknown.

Exosomes are nano-sized extracellular membrane-bound vesicles (30–100 nm) that could be involved in growth signal transmission across the RPE. All eukaryotic cells release exosomes from their endosomal compartments, except perhaps the mature erythrocytes (26). Studies have reported exosome biogenesis in a variety of bodily fluids (27), including tears and aqueous humor (28, 29). Although previously thought of as a means of cellular waste disposal, recent evidence points to the major physiological function of exosomes in mediating intercellular communication through the delivery of cargo to neighboring or distant cells (30, 31). Their cargo contains proteins, nucleic acids, and lipids unique to the cell of origin and can readily cross RPE tight junctions and retinal blood barriers (32), making them candidate growth signaling molecules. Proteomic evidence supports the biogenesis of exosomes from the RPE (33, 34), likely occurring on the apical side and mediated by the inhibition of G-protein coupled receptor (GPR)143 (35), which acts as a direct competitive antagonist receptor of dopamine—a potent myopia-protective neurotransmitter molecule (35, 36). Interestingly, the apical surface of the RPE also contains Na+/K+/ATPase, a known exosomal marker (37) whose expression levels have been linked to myopia (38, 39). In addition, the RPE apical surface is known to release several neurotransmitters, such as epidermal growth factor (40) and αβ crystalline (41, 42) that are implicated in the regulation of eye growth and refractive state.

These results lead to our hypothesis that exosomes released by the RPE may serve as candidate messengers to facilitate communication of growth signals from the neurosensory retina to the choroid. In this study, we provide preliminary evidence of *ex-vivo* RPE exosome biogenesis from the myopic and non-myopic tree shrew eyes. In addition, we demonstrate differential expression patterns of several RPE exosomal proteins in myopic eyes and highlight major cellular pathways by which RPE exosomes may facilitate growth signal transmission in the emmetropization mechanism.

## Methods

### Animals

Tree shrews (*Tupaia belangeri*) used in this study were raised by their mothers in the Tree Shrew Core at the University of Alabama at Birmingham. The colony is maintained on a 14-h light-on/10-h light-off cycle. Since tree shrews are born with their eyes closed, we designate the day of eye-opening (~3 weeks after birth) as the first day of visual experience (DVE). The age range of animals in this study was 156 to 210 DVE. All procedures were performed in adherence with the ARVO Statement for the Use of Animals in Ophthalmic and Vision Research and were approved by the Institutional Animal Care and Use Committee of the University of Alabama at Birmingham.

### Experimental groups

Nine tree shrews (five males/four females) were the subjects in this study. All animals within a group came from a different litter and were 156 to 210 DVE at the time of tissue collection. Individual eyes were categorized into myopic (*n* = 4 eyes) and non-myopic (control, *n* = 7 eyes) groups based on their non-cycloplegic refractive error. In myopic eyes, myopia was previously induced either by a −5 D lens or narrow-band cyan light, stimuli that are known to induce myopia in these animals (3, 43, 44). Non-myopic eyes were from animals raised in standard colony lighting who were near emmetropic after having completed their initial emmetropization process or had recovered from previous treatments to become near emmetropic. The average (mean ± SD) spherical equivalent refractive error (SER) was −9.26 ± 5.98 D for the myopic group and 0.45 ± 0.64 D for the non-myopic group (Table 1). The difference in SER between the groups was consistent with the difference in vitreous chamber depth (myopic: 3.05 ± 0.05 mm; non-myopic: 2.37 ± 1.04 mm).

**Table 1:** Characteristics of the experimental groups.

	**Myopic (*n* = 4)**	**Non-myopic (*n* = 7)**	*p*-value^*^
Days of Visual Experience (DVE, range)	159–210	156–210	
Right eye: left eye	3:1	4:3	
Male: female	3:1	3:4	
Spherical equivalent refractive error {D, mean [Sphere +(Cylinder/2)] ± SD}	−9.26 ± 5.98	0.45 ± 0.64	0.002
Vitreous chamber depth (mm)	3.05 ± 0.05	2.37 ± 0.10	0.0003

^*^Two sample t-test.

### Measurements of refractive error and ocular component dimensions

Non-cycloplegic refractive error was measured in awake and gently restrained animals in a dimly illuminated room using the Nidek infrared autorefractor (ARK-700A, Marco Ophthalmic, Jacksonville, FL, www.marco.com). To record these measurements, animals were aligned with the instrument using a pedestal installed on their skull, as described previously (45). A set of 10 measurements was taken, out of which five measurements with the highest quality scores were averaged to obtain the final SER. All refractive values were corrected for the “small eye artifact” (46) previously shown to be about +4 D in tree shrews (47). As with previous studies, we used non-cycloplegic data to quantify the SER because they have been shown to provide a valid estimate of refractive error in these species (48).

Following the measurement of refractive errors, axial ocular component dimensions were measured in awake and gently restrained animals with the LenStar (LS-900, Haag-Streit, www.haag-streit.com) using tree shrew-specific refractive indices (49). This optical biometer uses low-coherence interferometry to measure the dimensions of axial components. From these components, one can also calculate the vitreous chamber depth as the distance between the posterior lens surface and the internal limiting membrane of the retina. Three measurements were averaged to obtain the final measurement of axial components.

### RPE tissue preparation

**Figure 1:**
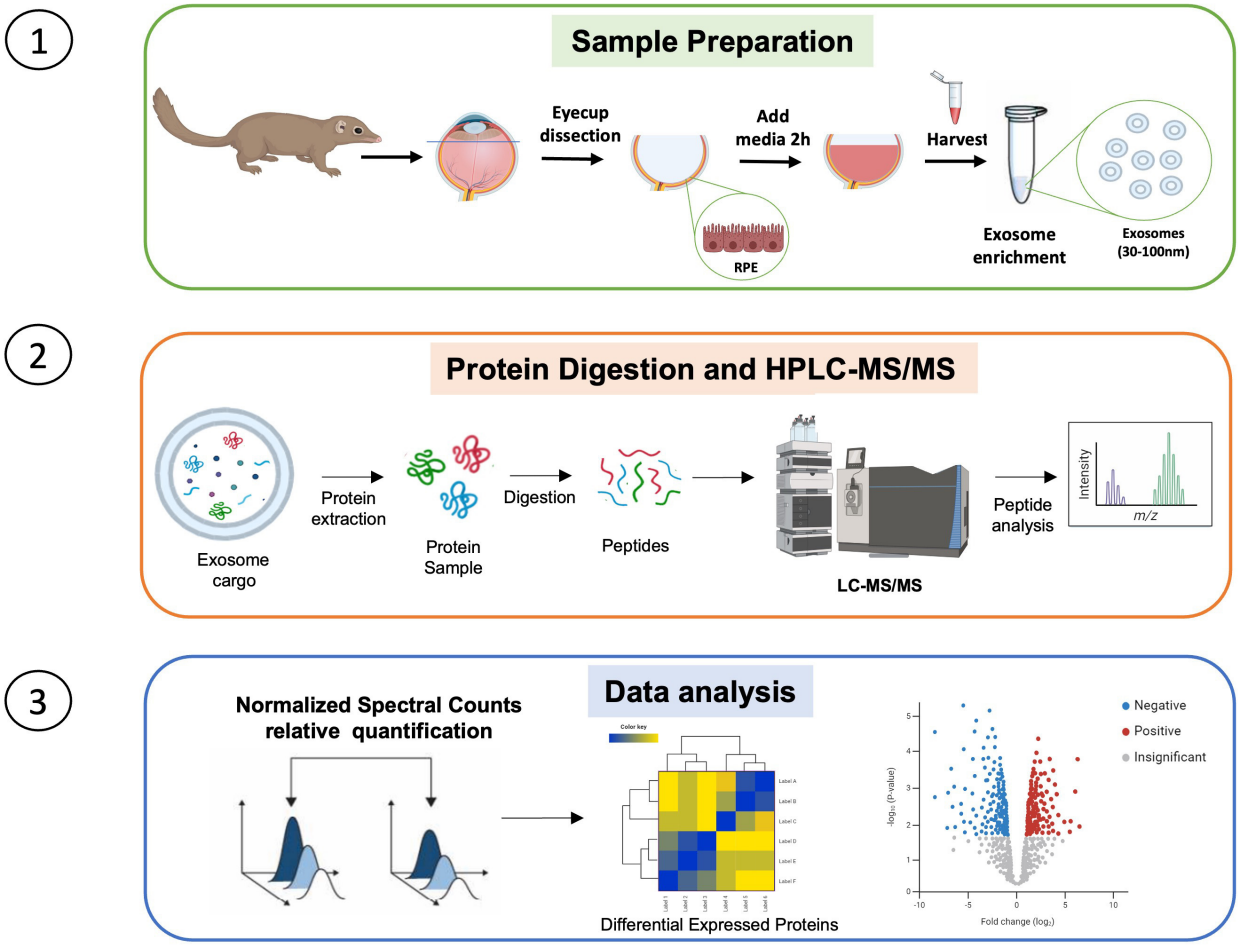
Graphical illustration of experimental methodology showing sample preparation, protein digestion and extraction, and data analysis. Created with BioRender.com.

After the animals were terminally anesthetized (17.5 mg ketamine and 1.2 mg xylazine, followed by 50 mg xylazine, intramuscular injection), eyes were enucleated and immediately put into a 10 cm Petri dish with sterile phosphate-buffered saline (PBS) for washing (Figure 1). Eyes were then transferred to a 6-well plate containing 4 mL of fresh PBS solution and dissected into an eyecup using a dissecting microscope and an 18 G needle. An incision was made in the sclera, ~1.0 mm behind the limbal boundary. Then, the anterior segment, including the cornea, iris, ciliary body, and crystalline lens, was removed. The retina was then detached from the remaining posterior segment eyecup, gently tugging on the zonule of Zinn, and then progressively peeled away, avoiding fragmentation. The eyecup with only RPE-choroid-sclera complex (RPE-lined eyecup) was washed twice with PBS and transferred to the upper chamber of 0.4 μm transwell insert of a 24-well plate (Corning, Cat# 3450, USA). Approximately 200 μl of L-15 media was added on top of the eyecup located in the upper chamber and 500 μl of media in the lower chamber of the insert. This allowed the eyecup to immerse in media, maintaining a normal flow of fluids on the RPE monolayer during incubation. The eyecup with media was then incubated at 37°C for 2 h, using a protocol modified from a previous report (35). After 2 h, the conditioning media was collected and centrifuged at 800 × g for 5 min at 4°C. The supernatant was then transferred to a new tube and stored at −80°C before enriching exosomes.

### Exosome enrichment and characterization

The RPE exosomes were isolated and purified using the double ultracentrifugation method (50). First, the eyecup conditioning media (~200 μl) was thawed and centrifuged at 1,000 × g for 10 min at 4°C. The supernatant was then transferred to an ultracentrifuge tube (#361623, for fixed angle rotor) and diluted to a volume of 4.5 mL using PBS before centrifuging the diluted sample at 10,000 × g for 30 min at 4°C using a fixed angle rotor. The supernatant was collected in an ultra-clear ultracentrifuge tube (#344057, for a swinging bucket rotor) and centrifuged in a swinging bucket rotor at 100,000 × g for 60 min at 4°C. After ultracentrifugation, the pellets were washed twice with 4.5 mL of PBS and briefly air-dried by flipping tubes upside down on Kimwipe (1–3 min). The excess PBS on the tube wall was wiped off using Kimwipe without disturbing the pellet/bottom of the tube. The pellet was then reconstituted in 40 μl of PBS by pipetting up and down ~20 times gently, and resuspended samples were prepared for quality checks and proteomic analysis.

Three phenotyping methods were used to characterize the enriched exosomes: transmission electron microscopy (TEM, Tecnai Spirit T12), CD63 enzyme-linked immunoassay, and nanoparticle tracking analysis (NTA), as recommended by the International Society for Extracellular Vesicles (51). The morphology of particles in the samples was visualized with transmission electron microscopy (TEM, Tecnai Spirit T12) after preparing the samples according to a previously published protocol (27). We also evaluated CD63 expression [a molecular marker of exosomes (27)] in the samples using ExoELISA-ULTRA CD63 Kit (#EXEL-ULTRA-CD63-1, System Biosciences, SBI) following the manufacturer's instructions.

### Nanoparticle tracking analysis

The size and concentration of exosomes released from RPE-lined eyecups were measured with NTA using NanoSight NS300 (Malvern Instruments Inc., Westborough, MA) equipped with a 488nm laser and integrated automated fluidics. Five 60-s videos were recorded of each sample with the camera level set at 13 and the detection threshold set at 5. The temperature was set at 25°C and monitored throughout the measurements. Videos recorded for each sample were analyzed with NTA software version 3.4.4 to determine the concentration and size of measured particles with corresponding standard errors. For analysis, auto settings were used for blur, minimum track length, and minimum expected particle size. The NanoSight system was calibrated with polystyrene latex microbeads of 50, 100, and 200 nm (Thermo Scientific Inc.) before analysis. Exosome samples were diluted at 1:50 in PBS and 1 mL was used for NanoSight analysis. Sterile PBS (Gibco #20012-027) was used as a diluent to avoid contaminating particles. Five measurement runs were performed for each sample and averaged to obtain the final data. Results of NTA were displayed as frequency distribution graphs showing the number of particles per milliliter. The concentration of particles was calculated to determine the mean ± SD number of exosomes in the myopic and non-myopic samples.

### Mass spectrometry

#### Sample preparation

Proteomics analysis was carried out as previously referenced with minor changes (52) under section 2.5 nLC-ESI-MS2 under Protein IDs for GeLC. All protein extracts were attained using M-PER™ Mammalian Protein Extraction Reagent (Thermo Fisher Scientific, Cat. # 78501) and quantified using Pierce BCA Protein Assay Kit (Thermo Fisher Scientific, Cat.# PI23225). As was experimentally determined, a set amount of protein per sample was diluted to 35 μL using NuPAGE LDS sample buffer (1 × final conc., Invitrogen, Cat.# NP0007). Proteins were reduced with DTT and denatured at 70°C for 10 min prior to loading everything onto Novex NuPAGE 10% Bis-Tris Protein gels (Invitrogen, Cat.# NP0315BOX) and separated (35 min at 200 constant V). The gels were stained overnight with a Novex Colloidal Blue Staining kit (Invitrogen, Cat.# LC6025). Following de-staining, each entire lane was cut into multiple MW fractions (3–8 fractions, as is experimentally determined to be optimal) and equilibrated in 100 mM ammonium bicarbonate (AmBc), each gel plug was digested overnight with Trypsin Gold, Mass Spectrometry Grade (Promega, Cat.# V5280) following manufacturer's instruction. Peptide extracts were reconstituted in 0.1% Formic Acid (FA)/ddH_2_O at 0.1 μg/μL.

#### Protein quantification

Peptide digests (8 μL each) were injected onto a 1,260 Infinity nHPLC stack (Agilent Technologies) and separated using a 75-micron I.D. × 15 cm pulled tip C-18 column (Jupiter C-18 300 Å, 5 microns, Phenomenex). This system ran in line with a Thermo Q Exactive HFx mass spectrometer, equipped with a Nanospray FlexTM ion source (Thermo Fisher Scientific), and all data was collected in CID mode. The nHPLC was configured with binary mobile phases that included solvent A (0.1%FA in ddH_2_O), and solvent B [0.1%FA in 15% ddH_2_O/85% Acetonitrile (ACN)], programmed as follows; 10 min at 5%B (2 μL/min, load), 90 min at 5%-40%B (linear: 0.5 nL/min, analyze), 5 min at 70%B (2 μL/min, wash), 10 min at 0%B (2 μL/min, equilibrate). Following each parent ion scan (300–1,200 m/z at 60k resolution), fragmentation data (MS2) was collected on the topmost intense 10 ions at 7.5K resolution. For data-dependent scans, charge state screening and dynamic exclusion were enabled with a repeat count of 2, repeat duration of 30 s, and exclusion duration of 90 s.

#### MS data conversion and searches

The XCalibur RAW files were collected in profile mode, centroided, and converted to MzXML using ReAdW v. 3.5.1. The mgf files were created using MzXML2Search (included in TPP v. 3.5) for all scans. The data were searched using SEQUEST (Thermo Fisher Scientific), which is set for three maximum missed cleavages, a precursor mass window of 20 ppm, trypsin digestion, variable modification C at 57.0293, and M at 15.9949 as a base setting, with additional post-translational modifications (ex: Phos, Ox, GlcNAc, etc.) that may be applied later as determined to be of importance experimentally. Searches were performed with a species-specific subset of the UniProtKB database.

#### Peptide filtering, grouping, and quantification

The list of peptide IDs generated based on SEQUEST search results was filtered using Scaffold (Protein Sciences, Portland Oregon). Scaffold filters and groups all peptides to generate and retain only high-confidence IDs while also generating normalized spectral counts (N-SCs) across all samples for relative quantification. The filter cut-off values were set with a minimum peptide length of >5 AAs, with no MH+1 charge states, with peptide probabilities of >80% C.I., and with the number of peptides per protein ≥2. The protein probabilities were set to a >99.0% C.I., and an false discovery rate (FDR) < 1.0. Scaffold incorporates the two most common methods for statistical validation of large proteome datasets, the FDR and protein probability (53–55). Relative quantification across experiments was performed via spectral counting (56, 57), and when relevant, spectral count abundances were normalized between samples (58).

### Data and statistical analysis

#### Proteomic data analysis

For proteomic data generated, two separate non-parametric statistical analyses were performed between each pair-wise comparison. These non-parametric analyses include 1) the calculation of weight values by significance analysis of microarray (SAM; cut off >|0.8| combined with, 2) *T*-Test (single tail, unequal variance, cut off *p* < 0.05), which are then sorted according to the highest statistical relevance in each comparison. For SAM (59, 60), whereby the weight value (W) is a statistically derived function that approaches significance as the distance between the means (μ1-μ2) for each group increases, and the SD (δ1-δ2) decreases using the formula, W=(μ1-μ2)/(δ1-δ2). For protein abundance ratios determined with N-SCs, we set a 1.5–2.0-fold change (FC) as the threshold for significance, determined empirically by analyzing the inner-quartile data from the control experiments using ln-ln plots, where the Pearson's correlation coefficient (R) is 0.98, and >99% of the normalized intensities fell between the set fold change. In each case, all three tests (SAM, *T*-test, or FC) needed to pass to be considered significant.

#### Systems analysis

The Gene Ontology (GO) assignments and pathway analysis were performed using the ShinyGO 0.77 online tool (61). The results were verified with other online tools: Database for Annotation, Visualization, and Integrated Discovery and g: Profiler online tool (62). In addition, functional annotation clustering and Kyoto Encyclopedia of Genes and Genomes (KEGG) pathway mapping were performed. Protein networks and interactomes were analyzed with the STRING 9.1 public database (63).

## Results

### Characterization of exosomes released by RPE-lined eyecups

**Figure 2:**
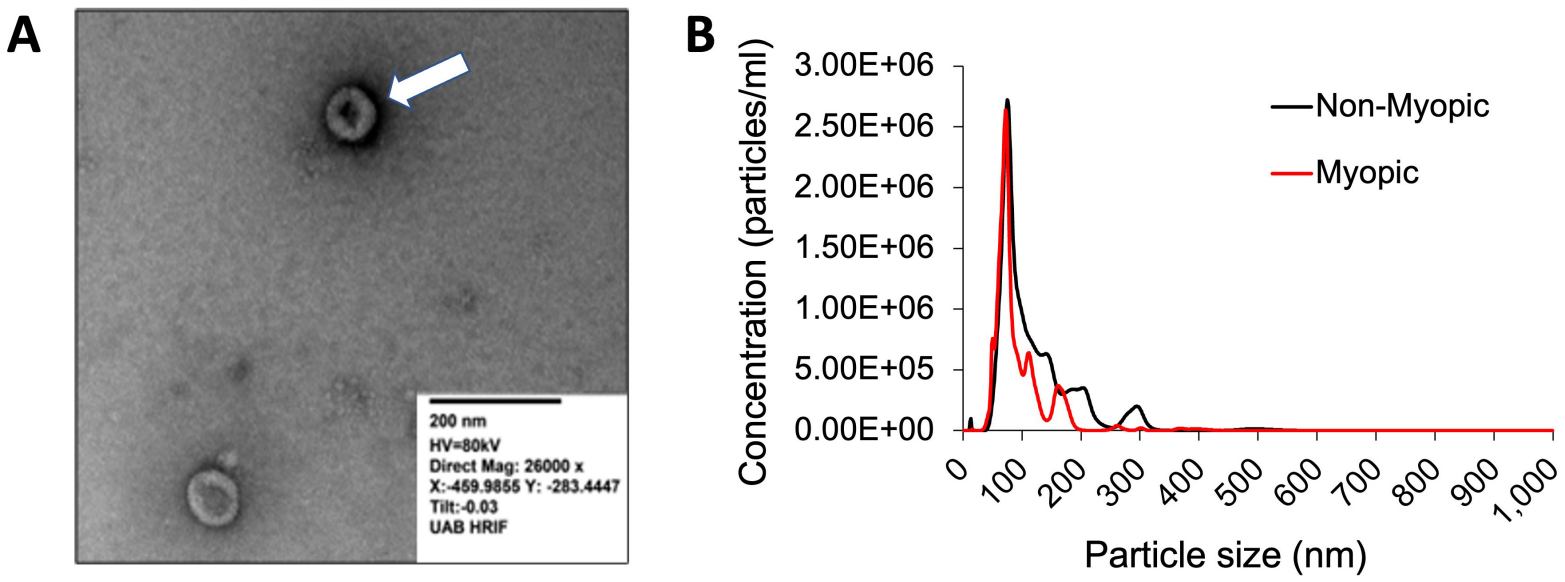
The phenotypic characterization of exosomes released by RPE-lined eyecups. **(A)** Homogeneous round-shaped membraned vesicles with diameters of 30–150 nm characteristic of exosomes were observed in transmission electron microscopy (scale: 200 nm). **(B)** Concentration of particles as a function of their size on Nanoparticle Tracking Analysis (NTA) of exosomes enriched samples: non-myopic (black line) and myopic (red line). The peak size of particles was 72.3 nm for non-myopic samples and 67.6 nm for myopic samples.

Figure 2 illustrates the phenotypic characterization of exosomes isolated from the RPE-lined eyecups of myopic and non-myopic tree shrew eyes. The TEM images of the samples showed homogeneous round-shaped membraned vesicles on exosome-enriched samples (Figure 2A). The particle size was in the range expected for exosomes (64) and peaked at 72.3 ± 2.3 nm for the non-myopic sample and 67.6 ± 2.3 for the myopic sample (Figure 2B). The size heterogeneity between the myopic and non-myopic samples may be related to biological roles, such as cellular processes, disease mechanisms, or cargo content and delivery (65, 66). The mean ± SD concentration of particles was 1.43 ± 0.06 × 10^8^ particles/mL in the non-myopic sample and 9.4 ± 0.37 × 10^7^ particles/mL in the myopic sample. The presence of the CD63, a molecular marker of exosomes (51), was measured by ExoELISA-ULTRA CD63 Kit (#EXEL-ULTRA-CD63-1, System Biosciences, SBI). The samples were positive for CD63, with a range of exosome abundance from 4 × 10^8^ to 7 × 10^8^ across samples.

### Proteomic profile of RPE exosomes from myopic and non-myopic eyes

**Figure 3:**
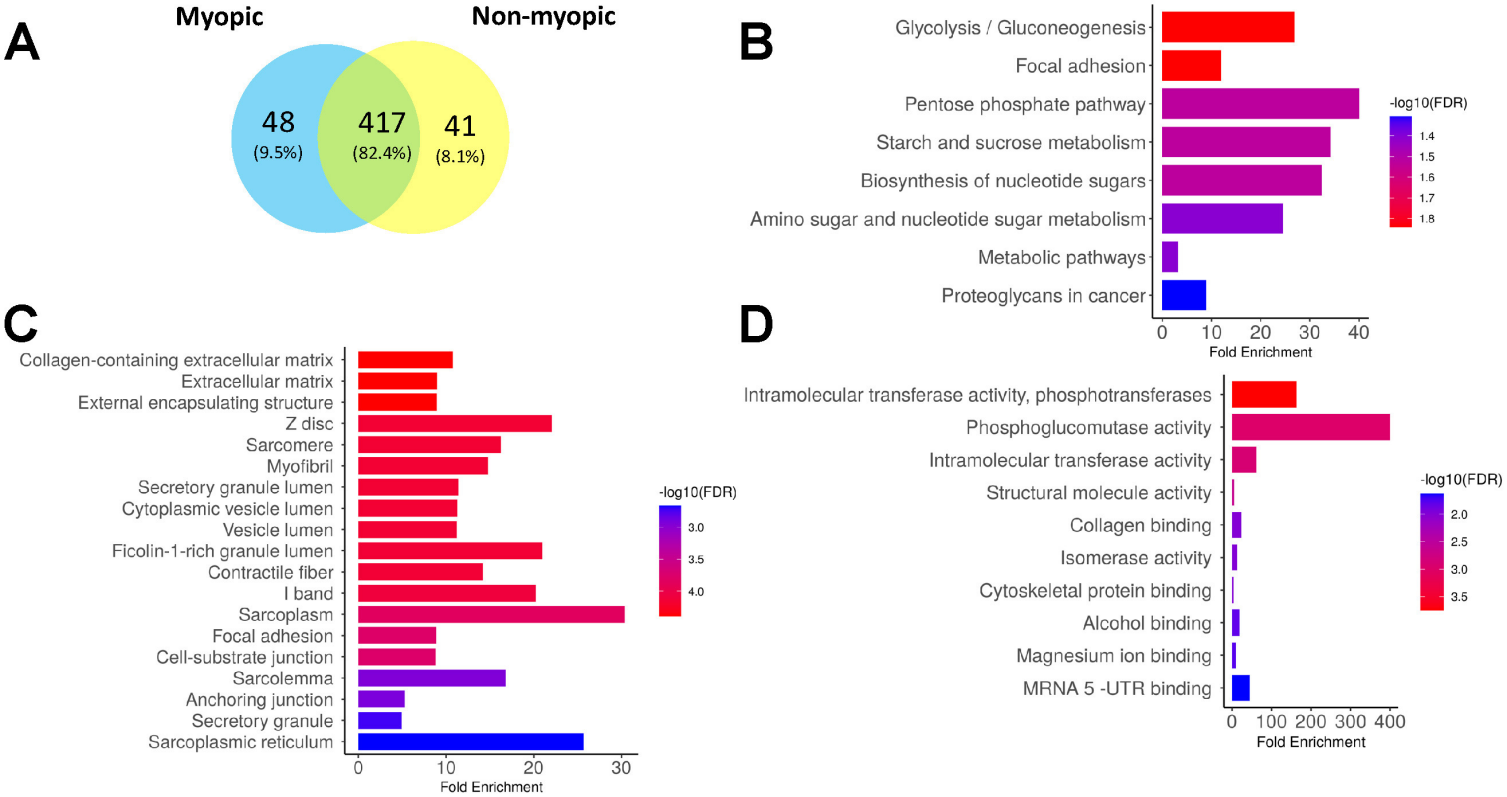
Proteomic profiles of RPE exosomes from myopic samples. **(A)** Venn diagram displaying all identified RPE exosomal proteins across the myopic and non-myopic samples. Enrichment analysis from KEGG **(B)**, GO cellular components **(C)** and GO molecular functions **(D)** for 48 unique RPE exosomal proteins identified in myopic samples. All enrichment analysis were performed with ShinyGo 0.77 online tool.

**Figure 4:**
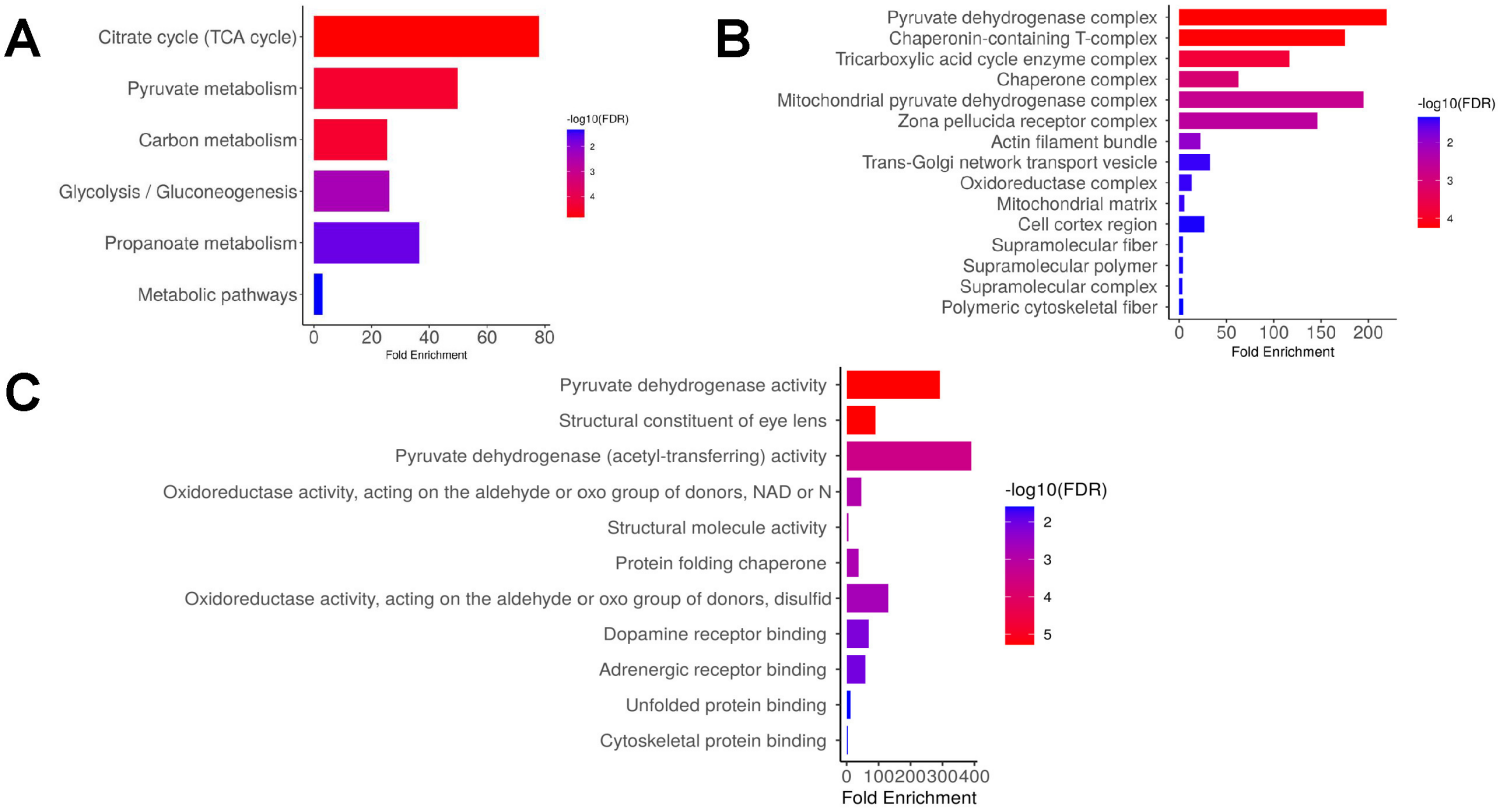
Proteomic profiles of RPE exosomes from non-myopic samples. Enrichment analysis from KEGG **(A)**, GO cellular components **(B)** and GO molecular functions **(C)** for 41 unique RPE exosomal proteins identified in non-myopic samples. All enrichment analysis were performed with ShinyGo 0.77 online tool.

A total of 506 proteins were identified across the myopic and non-myopic RPE exosome samples. Out of these, 417 were common, 48 were uniquely expressed in the myopic samples and 41 were uniquely expressed in the non-myopic samples (Figure 3A; Supplementary Tables S1, S2). The enrichment analysis for KEGG, GO cellular components, and GO molecular functions performed on ShinyGO 0.77 for uniquely expressed proteins are shown in Figures 3B–D for myopic samples and Figures 4A–C for non-myopic samples. The RPE exosomal proteins uniquely expressed in myopic samples were linked to the metabolism of carbohydrates and amino sugars with phosphoglucomutase and phosphotransferase molecular activity which contribute to the upregulation of glycolysis pathway in target cells (Supplementary Table S1). Other notable GO cellular components associated with myopic RPE exosomal proteins were extracellular matrix, intracellular vesicles, and focal adhesion and paracrine factors related to extracellular matrix remodeling. Examples of identified proteins in these categories were Heat shock protein family B (small) member 1(HSPB1), Transforming growth factor-beta-induced protein (TGFBI), Myocilin (MYOC), Apolipoprotein A-IV (APOA4), Thrombospondin-1 (THBS1), protein phosphatase 1 catalytic subunit beta (PPP1CB), Myosin binding protein C2 (MYBPC2), Phosphoglucomutase-like protein 5 (PGM5), and Collagen type VI alpha 3 chain (COL6A3; Supplementary Tables S1, S2).

In the non-myopic samples, the uniquely expressed proteins were involved in the metabolism of tricarboxylic acid and pyruvate (Figure 4A). In addition, mitochondrial components with pyruvate dehydrogenase enzymatic activity, chaperone complex, and tricarboxylic acid cycle (TCA) enzymes were the most enriched cellular components (Figures 4B, C). Examples of identified proteins in these categories were Dihydrolipoyl dehydrogenase, mitochondrial (DLD), Pyruvate dehydrogenase E1 component subunit alpha (PDHA1), Succinyl-CoA ligase [ADP-forming] (SUCLA2), T-complex protein 1 subunit beta (TCPB), Beta-crystallin A2, A3, B1, B3 (CRYBA2, CRYBA3, CRYB1 and CRYB3; Supplementary Tables S3, S4). These analyses suggest that the proteomic profile of RPE exosomes from myopic eyes shows a differential expression pattern, which likely supports altered metabolic requirements of the myopic retina and extracellular matrix remodeling process.

### Identification of proteins differentially expressed in RPE exosomes from myopic eyes

**Figure 5:**
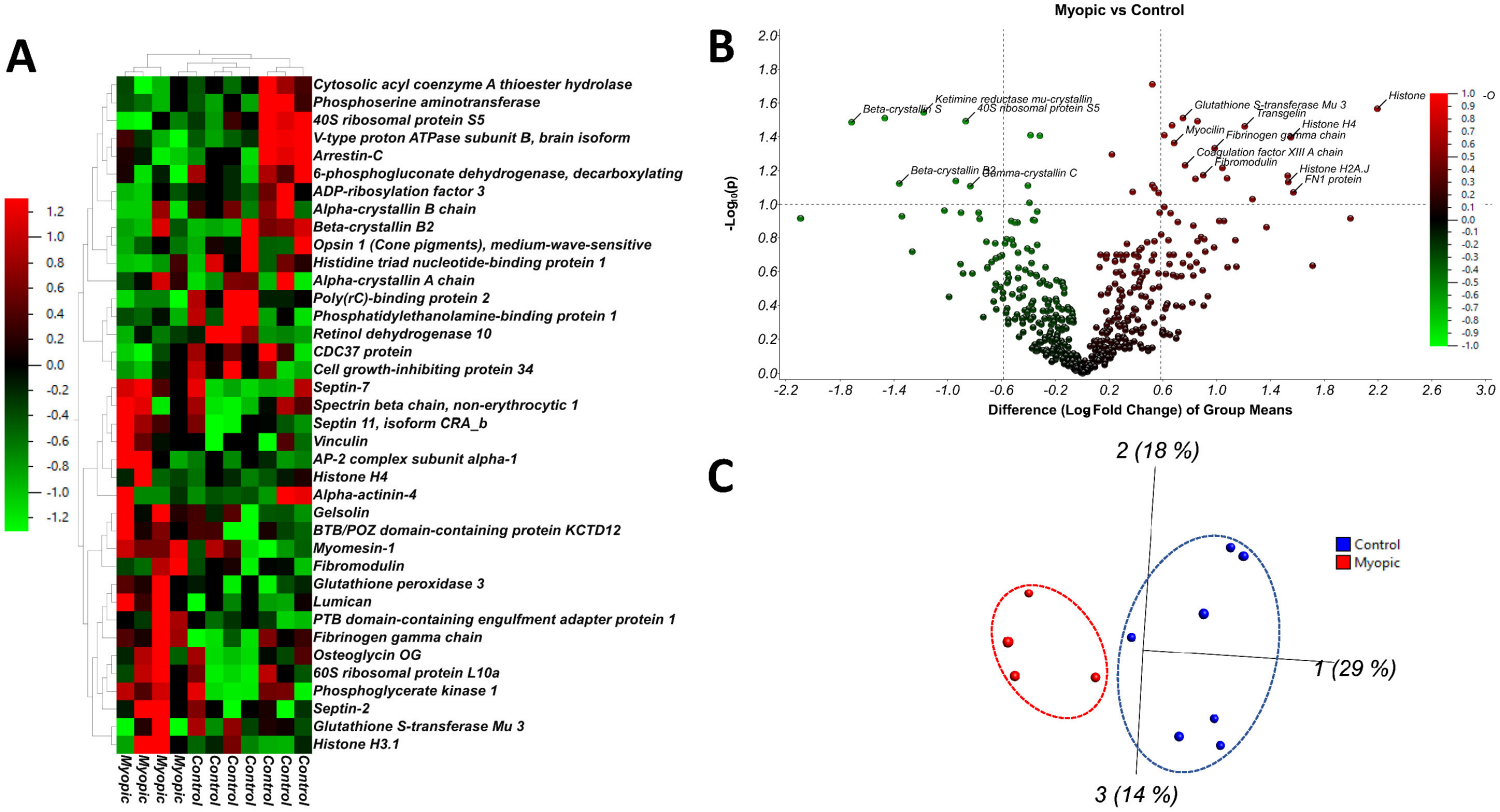
Differential expression patterns of top 38 RPE exosomal proteins in myopic eyes. **(A)** Heatmap of these proteins comparing expression levels between myopic and non-myopic (control) eyes. The box color indicates log_2_ fold changes showing upregulation in red and downregulation in green. **(B)** Volcano plot illustrating top 40 significantly differentially abundant proteins selected by Qlucore Stats. The –log_10_ (Benjamini–Hochberg corrected *P*-value) is plotted against the log_2_ (fold change, FC: myopic/non-myopic). The non-axial vertical lines denote ±1.5-fold change while the non-axial horizontal line denotes *p* = 0.05, which is the significance threshold (prior to logarithmic transformation). Volcano plot shows significantly upregulated proteins with red dots (FC≥1.5) into the right of the non-axial vertical right line and significantly downregulated proteins as green dots (FC≤-1.5) into the left of the non-axial vertical line. **(C)** 3D Principal component analysis (PCA) shows that myopic (red) and non-myopic (control, blue) samples are well distinguished by two defined clusters.

The comparison of proteomic profiles of RPE exosomes between myopic and non-myopic eyes exhibited 286 differentially expressed proteins. Out of these, 79 were significantly upregulated and 70 were significantly downregulated (Supplementary Tables S5, S6). Table 2 summarizes the top 41 differential expressed proteins, including 21 upregulated, 16 downregulated, and 3 undetected in myopic samples. The heatmap of these proteins showed several protein clusters that were upregulated and downregulated in myopic samples compared with non-myopic samples (Figure 5A).

**Table 2:** Differentially expressed RPE exosomal proteins in myopic eyes.

**Protein names**	**Accession number**	**Gene symbol**	**SAM**	**T-test**	**Fold Change (Myopic/Non-myopic)**	**Expression level in myopic eyes**
Histone H4	P62805	H4C9	0.686	0.098	3.04	Upregulated
PTB domain-containing engulfment adapter protein 1	Q9UBP9	GULP1	1.036	0.076	2.59	
Histone H3.1	P68431	H3C10	0.626	0.137	2.59	
AP-2 complex subunit alpha-1	O95782	AP2A1	0.863	0.107	2.43	
Lumican	P51884	LUM	1.445	0.014	2.27	
Vinculin	P18206	VCL	0.693	0.092	2.22	
Septin 11, isoform CRA_b	D6RGI3	SEPTIN11	0.937	0.036	2.19	
Glutathione peroxidase 3	P22352	GPX3	0.921	0.058	2.12	
Spectrin beta chain, non-erythrocytic 1	Q01082	SPTBN1	0.730	0.030	1.94	
Septin-7	Q16181	SEPTIN7	0.882	0.014	1.91	
60S ribosomal protein L10a	P62906	RPL10A	0.579	0.090	1.84	
BTB/POZ domain-containing protein KCTD12	Q96CX2	KCTD12	0.715	0.070	1.82	
Fibrinogen gamma chain	P02679	FGG	0.828	0.014	1.80	
Osteoglycin OG	Q7Z532	OGN	0.498	0.094	1.79	
Fibromodulin	Q06828	FMOD	0.580	0.100	1.73	
Phosphoglycerate kinase 1	P00558	PGK1	0.683	0.032	1.70	
Septin-2	Q15019	SEPTIN2	0.780	0.038	1.70	
Alpha-actinin-4	O43707	ACTN4	0.491	0.097	1.65	
Glutathione S-transferase Mu 3	P21266	GSTM3	0.875	0.018	1.64	
Gelsolin	P06396	GSN	0.793	0.034	1.57	
Myomesin-1	P52179	MYOM1	0.918	0.007	1.50	
Cell growth-inhibiting protein 34	Q08ES8	RPL11	−0.677	0.048	−1.53	Downregulated
Arrestin-C	P36575	ARR3	−0.487	0.100	−1.57	
V-type proton ATPase subunit B, brain isoform	P21281	ATP6V1B2	−0.452	0.087	−1.59	
Alpha-crystallin A chain	P02489	CRYAA	−0.644	0.031	−1.61	
CDC37 protein	Q6FG59	CDC37	−0.810	0.042	−1.64	
Phosphoserine aminotransferase	Q9Y617	PSAT1	−0.581	0.072	−1.70	
Alpha-crystallin B chain	P02511	CRYAB	−0.529	0.071	−1.75	
6-phosphogluconate dehydrogenase, decarboxylating	P52209	PGD	−0.625	0.042	−1.79	
Phosphatidylethanolamine-binding protein 1	P30086	PEBP1	−0.525	0.069	−1.80	
Opsin 1 (Cone pigments), medium-wave-sensitive	B7ZLG5	OPN1MW	−0.526	0.067	−1.81	
Histidine triad nucleotide-binding protein 1	P49773	HINT1	−0.530	0.060	−1.83	
Retinol dehydrogenase 10	Q8IZV5	RDH10	−0.487	0.078	−1.86	
Poly(rC)-binding protein 2	Q15366	PCBP2	−1.174	0.006	−2.10	
ADP-ribosylation factor 3	P61204	ARF3	−0.661	0.073	−2.12	
Beta-crystallin B2	P43320	CRYBB2	−0.628	0.051	−2.14	
Cytosolic acyl coenzyme A thioester hydrolase	O00154	ACOT7	−0.745	0.045	−2.15	
40S ribosomal protein S5	P46782	RPS5	−1.246	0.004	−2.41	
Beta-crystallin A2	P53672	CRYBA2				Undetectable
Beta-crystallin A3	P05813	CRYBA1				
Beta-crystallin B3	P26998	CRYBB3				

A volcano plot was constructed to display the top 41 differentially expressed proteins (Figure 5B). Above the horizontal threshold line (*p* = 0.05), the significantly upregulated proteins (≥1.5 FC, myopic/non-myopic) are shown as red dots and the significantly downregulated proteins (≤ -1.5 FC, myopic/non-myopic) are shown as blue dots. Out of the differentially expressed proteins, the most abundant proteins in the myopic samples were Histone H4 (H4C9, FC = 3.04), PTB domain containing engulfment adaptor protein 1 (GULP1, FC = 2.59), Histone H3.1 (H3C10, FC = 2.59), Lumican (LUM, FC = 2.27), Vinculin (VSL, FC = 2.2), Septin-2,7,11 (SEPTIN 2,7,11, FC = 1.70 to 2.19), Gelsolin (GSN, FC = 1.57), Alpha-actin 4 (ACTN4, FC = 1.65), Fribomodulin (FMOD, FC = 1.73), and Fibrinogen gamma chain (FGG, FC = 1.80). Similarly, the least abundant proteins were Cell growth-inhibiting protein 34 (FC = 1.53), Arrestin-C (FC = −1.57), Alpha-crystallin A and B chains (FC = −1.61 to −1.75), Opsin 1 medium-wave-sensitive (FC = −1.81), Retinol dehydrogenase 10 (FC = −1.86), Poly(rC)-binding protein 2 (FC = −2.10), 40S ribosomal protein S5 (RPS5, FC = −2.41), Cytosolic acyl coenzyme A thioester hydrolase (ACOT7, FC = −2.15) and Beta-crystallin B2 protein (CRYBB2, FC = −2.14) (Figure 5B and Table 2). The principal component analysis of the exosome protein data showed two well-defined clusters for the myopic and non-myopic groups (Figure 5C).

### Functional analysis of the top 41 differentially expressed RPE exosome proteins in the myopic eyes

**Figure 6:**
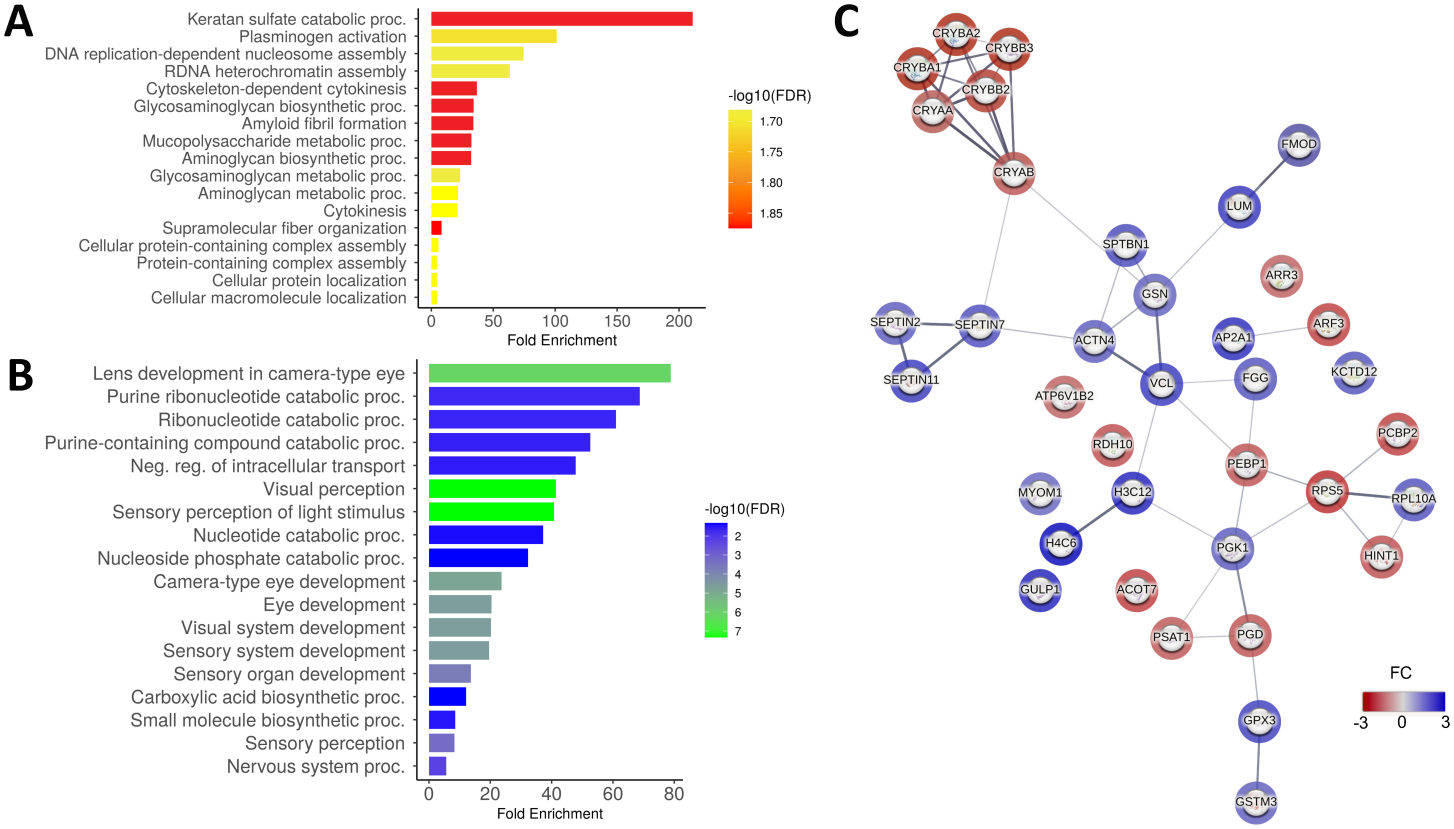
Enrichment analyses and networks interactions of top 41 differentially expressed RPE exosomal proteins in the myopic eyes. GO analysis for biological processes of 21 significantly upregulated proteins **(A)** and 17 significantly downregulated proteins and 3 undetected proteins **(B)** in myopic samples performed with the ShinyGO 0.77 online tool. **(C)** Protein–protein interaction network on STRING database of top 41 differentially expressed proteins. Fold changes are in border color of the circles: upregulated proteins in blue and downregulated proteins in red.

The functional GO analysis of the top 41 differentially expressed proteins showed that the upregulated RPE exosomal proteins in myopic eyes were closely related to cytokinesis, glycan metabolism, and nucleosome activation pathways, including some previously reported in eye growth regulation, such as oxidative stress (67), TGF-β receptor signaling (68), and plasminogen activation (69) (Figure 6A). The most significant biological process was the response to lipid hydroperoxide. The results suggested that the upregulated protein groups could be involved in the cytoskeleton organization mediated by SEPTIN proteins and increased resistance to remodel the extracellular matrix (Supplementary Figure S1B). On the contrary, the downregulated RPE exosomal proteins in myopic eyes were mostly related to lens development, visual perception, sensory system development, and nucleotide catabolism (Figure 6B). Although the KEGG and GO analyses for the downregulated proteins in myopia did not show results in ShinyGO sorting for cellular components, these proteins were found to be related to the structure of the eye lens when they were sorted by molecular functions (Supplementary Figure S1D).

The ingenuity pathway analysis by category of diseases and biomarkers of 41 differentially expressed RPE exosomal proteins in myopic samples showed 35 members altered in cancer, organismal injury, and abnormalities, 23 in neurological diseases, and 13 members involved in ophthalmic diseases including conditions related to abnormal morphology of the eye (Supplementary Table S7). The top canonical pathways for differentially expressed RPE exosomal proteins in myopia were related to integrin signaling, Ras homolog family member A (RHOA) signaling, cell junction signaling, and phototransduction pathway (Supplementary Table S8).

### An interaction network model for differentially expressed RPE exosomal proteins in myopia eyes

To identify the candidate protein networks involved in myopia, we used the top 41 differentially expressed RPE exosomal proteins to construct a protein-protein interaction (PPI) network model using the STRING database where nodes were defined with a score ≥ 0.4 (Figure 6C). The border color indicates fold change of differentially expressed proteins in myopic samples compared with non-myopic samples: upregulated proteins in blue and downregulated proteins in red. Closer connectivity between each node was represented by shorter distances and the number of connective lines that joined each circle. The connectivity was the most pronounced in the SEPTIN and Beta-crystalline protein interaction groups (Figure 6C).

### Identification of potential RPE exosome protein biomarkers of myopia

**Figure 7:**
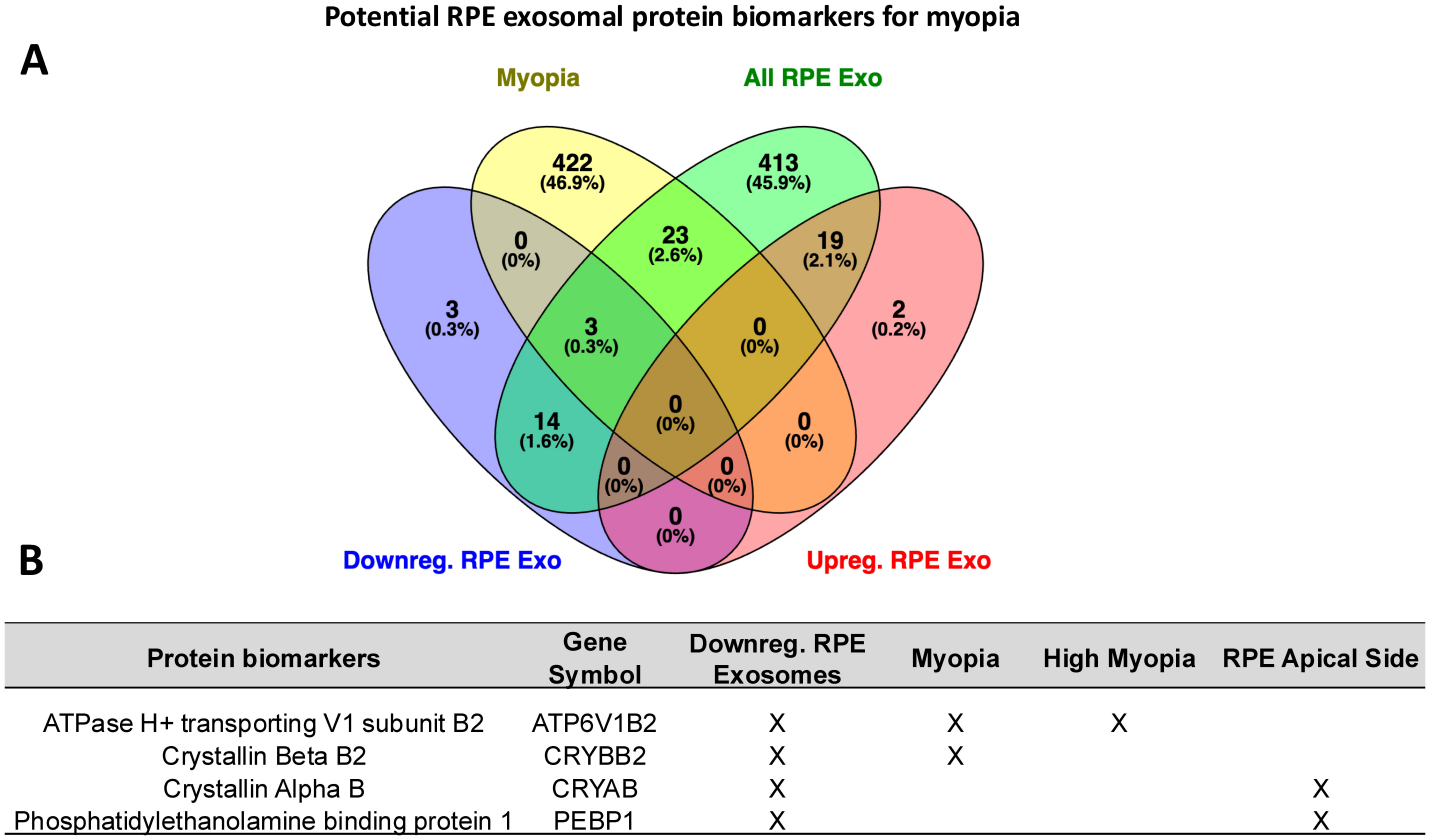
Profile of RPE exosomal proteomics compared with published data. **(A)** Venn diagram showing significantly downregulated RPE exosomal proteins in myopic samples (blue), significantly upregulated RPE exosomal proteins in myopic samples (red), and all identified RPE exosomal proteins (green) in this study compared with all myopia monarch proteins listed on STRING database (yellow). **(B)** Common proteins between the current study and published data: all myopia monarch and high myopia proteins listed on STRING database, and RPE apical side-released proteins reported previously (33).

We also compared the proteomic profile of RPE exosomes from myopic eyes with data deposited on the STRING database for myopia (448 genes), high myopia (41 genes) (63), and previously reported proteomic data from the apical side of the RPE (55 proteins) (33). We found 23 common proteins with the STRING database (Figure 7A), although these proteins did not show significant expressions on myopic samples. Three significantly downregulated RPE exosomal proteins in our myopic samples were common with the previous reports: ATPase H+ transporting V1 subunit B2 (ATP6V1B2), Crystallin beta B2 (CRYBB2), and Arrestin 3 (ARR3) (Figure 7A). Proteins ATP6V1B2 (70), CRYBB2 (68), and ARR3 (71) have been previously associated with myopia and/or high myopia (Figure 7B), suggesting that myopia could be linked with reduced expression levels of these RPE exosomal proteins. Two other previously reported RPE-derived apical exosomal proteins (33), Phosphatidylethanolamine binding protein 1 (PEPB1) and Crystallin alpha B (CRYAB) (72), were significantly downregulated in our myopic samples (Figure 7B and Supplementary Figure S2B). Protein CRYAB was also downregulated in an animal model of myopia induced by form deprivation (72).

## Discussion

In this preliminary study, we provided the first demonstration of RPE exosome biogenesis in tree shrew eyes using an innovative *ex vivo* model. By characterizing the proteomic profiles of exosomes released by the RPE in myopic and non-myopic eyes, we further showed that myopia is associated with proteomic alterations in RPE-derived exosomes. Additionally, we identified several RPE exosomal proteins as candidate biomarkers that could play a role in mediating growth signal communication across the RPE in the control of eye growth and refractive state.

There is prior evidence that proteomic profiles of exosomes are altered in myopia (28, 73, 74). However, these studies quantified protein expression levels in exosomes derived from ocular fluids such as aqueous or vitreous humor, so they are unlikely to represent exosomal characteristics and changes involved in the retina-choroid-sclera growth signaling pathway of emmetropization. The RPE is a critical mediator of growth signals between the neurosensory retina and the choroid (18, 20). It has been shown that exosomes derived from the RPE contain numerous signaling proteins in normal physiological conditions (33) including oxidative stress-induced signaling phosphoproteins (34). The RPE monolayer can potentially serve as receptors of putative signaling molecules like dopamine, retinoic acid, and adenosine and mediators of associated growth signals into the choroid (15, 36). Therefore, the mechanistic investigation of RPE-derived exosomes and exosomal proteomic profiles could provide insights into potential protein biomarkers involved in intracellular signaling of growth information in the emmetropization mechanism.

### Exosomal proteins secreted by the RPE exclusively in myopic eyes

Among the 48 RPE exosomal proteins uniquely expressed in myopic eyes, we identified several family members of previously reported cytoskeletal and structural proteins related to myopia: Myosin-binding protein C (MYBPC) and Tropomyosin 1α4 (TPM4) (76), extracellular matrix proteins like Collagen alpha 3(VI) chain (COL6A3) (75), and Apolipoproteins A-IV and B (APOA4 and APOB) (74, 76). Additionally, this group of uniquely expressed RPE exosomal proteins in myopia included Thrombospondin 1 (THBS1), Keratocan (KERA), and Myocilin (MYOC) that were reportedly downregulated in the sclera of myopic tree shrew eyes (70). Considering that the same set of proteins was observed both in the RPE and sclera, the translocation of these proteins might facilitate growth signal transmission to affect the extracellular matrix remodeling in the sclera (77). Indeed, the cellular components of uniquely expressed myopic RPE exosomal proteins were primarily related to extracellular matrix, intracellular vesicles, and focal adhesion, indicating a potential role in extracellular matrix remodeling. These results suggest that the RPE exosomes likely serve as a facilitator of cellular communication to trigger morphological changes in the sclera.

### Exosomal proteins secreted by the RPE exclusively in non-myopic eyes

The proteomic analysis revealed 41 RPE exosomal proteins uniquely expressed in non-myopic eyes. These proteins may have a role in the homeostatic maintenance of growth inhibitory STOP signals to produce optimal “physiological” eye growth (78, 79). For example, the unique expression of Beta-arrestin-2 (ARRB2) in non-myopic eyes is consistent with its role in the regulation of dopamine D2 receptor activity. It has been shown that ARRB2 is involved in the desensitization and internalization of dopamine D2 receptors (137) and that the activation and inactivation of these receptors could lead to myopia development and inhibition, respectively (138, 139).

Three members of the crystallin β family—CRYBB3, CRYBA2, and CRYBA1—were exclusively expressed in the non-myopic samples. These crystallin proteins are part of the α, β, and γ crystallin superfamily, which plays a crucial role in maintaining the transparency of the crystalline lens. Interestingly, members of this protein superfamily are also found in posterior ocular structures, such as the RPE, photoreceptor inner and outer segments, and the outer nuclear layer of the retina (140, 141). Studies have shown that these crystallin proteins in the RPE respond to light intensity (142) and oxidative stress (143), similar to the αβ crystallins in the lens. Furthermore, the presence of these crystallin proteins in non-myopic eyes aligns with previous findings of αβ crystallins in exosomes derived from the apical surface of human RPE cells (42). These crystallins were also localized in the interphotoreceptor matrix, suggesting their uptake from the extracellular space as part of a protective response to oxidative damage and neural stress (42). Additionally, there is evidence of signaling interactions between the lens and the posterior segment (80–82). Collectively, these findings imply that the crystallins observed could play a role in exosome-mediated intercellular communication between photoreceptors and the RPE within the emmetropization pathway.

### Upregulation of RPE exosomal proteins in myopic eyes

In myopic eyes, the RPE exosomes exhibited higher levels of PTB domain-containing engulfment adapter protein 1 (GULP1) and Adaptor-related protein complex 2 (AP2A1) compared to non-myopic eyes. GULP1 is essential for phagocytosis of apoptotic cells, transport of glycosphingolipids and cholesterols, and endosomal trafficking of various low-density lipoprotein receptor-related protein 1 (LRP1) ligands (83). In contrast, AP2A1, a subunit of the adaptor protein complex 2 (AP-2), plays a role in clathrin-mediated endocytosis and helps facilitate the internalization of LRP1 and its ligands by promoting the formation of clathrin-coated vesicles (84). LRP1 functions as a scavenger of tissue inhibitors of metalloproteinases (TIMP), both of which are found in the RPE (144, 145). The upregulation of GULP1 and AP2A1 could lead to greater removal of TIMP by LRP1 in the RPE cells (85), promoting transforming growth factor (TGF)-β activation and oxidative stress and potentially contributing to myopia development (146). LRP1 deficiency has been shown to be associated with myopia phenotype through TGF-β activity (86, 87).

The RPE exosomes of myopic eyes showed upregulation of several structural proteins: actin filament binding protein, Vinculin (VCL); a filament-forming cytoskeletal GTPase, Septin family proteins (SEPTIN2, SEPTIN 7, and SEPTIN11); and focal adhesion cytoskeleton protein, βII spectrin (SPTBN1). Vinculin and βII spectrin proteins are known to be involved in cell-cell adhesion (88, 89). Additionally, Vinculin regulates cell-matrix adhesion and E-cadherin expression on cellular surfaces and potentiates mechanosensing by the E-cadherin complex (88). SEPTIN11 promotes cell motility and cell adhesion by activating the RhoA protein (90) and the Septin protein family plays a potential role in cytokinesis (91). Other upregulated RPE exosomal proteins in myopic eyes were Lumican (LUM) and Fibromodulin (FMOD), closely related members of the extracellular matrix leucine-rich repeat glycoprotein/proteoglycan family. These proteins bind to fibrils allowing assembly of the collagen network in the extracellular matrix (92). LUM is a keratan sulfate proteoglycan that promotes fibroblast-myofibroblast transition. It has been reported to increase transcription of alpha-smooth muscle actin, matrix metallopeptidase 9, Collagen I, plasminogen activator inhibitor 1, and TGF-β *in vitro* (93), and play several roles in ocular diseases (94). LUM helps to maintain stability and tension of the extracellular matrix in the sclera by interacting with collagen fibers (95). This interaction of LUM with collagen fibers could be involved in myopiagenesis as its gene mutation and polymorphism have been associated with myopia in humans (96) and animal models (97). For example, LUM mutant zebrafish eyes exhibited ocular enlargement primarily due to disruption of collagen fibril arrangement leading to scleral thinning and reduced stiffness (97). Additionally, double knockouts of LUM and FMOD were found to cause axial eye elongation, retinal detachment, and scleral thinning, suggesting that both proteins are critical for scleral ensemble and functioning and may underlie morphological changes in the sclera of myopic eyes (98). The proteoglycans discussed in this section have also been identified in various retinal layers, including the RPE and interphotoreceptor matrix (147). Therefore, the increased levels of these proteins in RPE exosomes from myopic eyes may indicate a compensatory response to the heightened potency of GO signals in the early emmetropization pathway, or a protective mechanism aimed at maintaining the integrity of RPE, interphotoreceptor matrix, and other retinal layers.

Proteomic analyses of RPE exosomes in myopic eyes also showed an upregulation of Alpha Actinin-4 (ACTN4) and Gelsolin (GSN), proteins previously linked with the biological basis of myopia (76). These results suggest that cytoskeletal structural proteins and coagulation pathways may have a role in myopia development. Another highly expressed exosomal protein in the myopic eyes was glutathione S-transferase mu 3 (GSTM3), a potent antioxidant enzyme that reduces glutathione and prevents neurotoxicity by cellular oxidative stress (99). It has been found that the suppression of the GSTM3 gene is associated with age-related cataract formation by oxidative stress in the lens (100). An overexpression of GSTM3 on myopic exosomes could be part of the neuroprotective response of RPE cells to counter feedback from the oxidative stress loop triggered by TGF-β (101), which is a known growth factor involved in myopiagenesis (68).

The upregulated RPE exosomal proteins were mainly involved in keratan sulfate catabolism, plasminogen activation, cytokinesis, and glycosaminoglycan and aminoglycan metabolism. The enhancement of these metabolic pathways is consistent with the reduced expression of glycosaminoglycans and proteoglycans in the sclera of myopic eyes (102, 103). Moreover, plasminogen protein has been proposed as a molecular marker of high myopia in humans (69). Proteins Fibromodulin (FMOD), Fibrinogen beta chain (FGB), and Fibrinogen gamma chain (FGG), which were upregulated in myopic eyes in this study, have also been reported to be upregulated in the retina of myopic rabbits (104). Since fibrinogen stimulates tissue plasminogen activators against plasminogen, (105) and plasminogen activators, like matrix metalloproteases, are mediators of extracellular proteolysis (106), they could act in similar ways to produce extracellular matrix remodeling in myopia.

### Downregulation of RPE exosomal proteins in myopic eyes

The RPE of myopic eyes released exosomes with lower levels of proteins encoded by genes whose mutations are reportedly linked with myopia, such as Opsin 1 medium-wave-sensitive (OPN1MW) (107–109), Arrestin-C (ARR3) (71), α crystallin A and B (72, 110), and Retinol dehydrogenase 10 (RDH10) (111). Opsins are light-activated G-protein coupled transmembrane receptors that allow activation of the phototransduction pathway (112). These visual pigments contribute to human color vision (113). Opsin gene deficiency is related to color blindness (114) and its protein dysfunction is associated with cone dystrophy and myopia (108, 115, 116). ARR3 or cone-arrestin is a G-protein-coupled receptor that binds to phosphorylated opsins, after activation of the phototransduction pathway (117). ARR3 gene mutations are associated with high myopia (118). While the lower abundances of OPN1MW and ARR3 proteins in RPE exosomes of myopic eyes could indicate potential retinal dysfunction in myopia (119), it is unlikely that these proteins are packaged by the RPE cells into exosomes. Rather, exosomes that originate from cone photoreceptors are the likely source of these proteins. Presumably, the photoreceptor-derived exosomes were attached to the apical surface of the RPE and were harvested with RPE-derived exosomes.

α crystallin proteins were also downregulated in RPE exosomes of myopic eyes. These proteins belong to the small heat shock protein (HSP20) family and act as molecular chaperones. Downregulation of CRYAA mRNA and protein has been reported in high-myopic patients (110). CRYAB is known to be secreted by exosome-dependent pathways from polarized human RPE cells; they play a protective function in the interphotoreceptor matrix and confer resistance to heat and oxidative stress in cells (120, 121). Oxidative stress of retinal cells associated with myopia may initiate a downregulation of CRYAA and CRYAB secretion in the RPE exosomes. Similar results have been reported in the retina of an experimental glaucoma model, which showed significantly reduced expression of CRYAB (122). Another member of the crystallin protein family, CRYBB2, was found to be downregulated in our myopic samples. Notably, CRYBB2 has also been previously shown to be secreted in exosomes from the apical side of the RPE (33). Like other crystallin proteins, CRYBB2 has been demonstrated to help protect the RPE against oxidative stress (141). The reduced levels of crystallin proteins in RPE exosomes from myopic eyes may suggest a diminished capacity to respond to oxidative stress, potentially contributing to the dysregulation of eye growth and the development of myopia.

Another downregulated exosomal protein, RDH10, is a member of the retinol dehydrogenases family. RDH10 is mainly expressed in photoreceptors and RPE cells and reduces all-trans-retinal to all-trans-retinol during phototransduction (123). The RPE contains metabolic enzymes required for retinal signaling and metabolites like 11-cis-retinal that play a key role in the retinoid visual cycle (124). Retinoic acid, a metabolic precursor of vitamin A, has been implicated in ocular growth (15, 125) and is highly expressed in the myopic retina (126–128). It has also been shown that the extracellular matrix remodeling process in myopia could be regulated by the cell differentiation function of retinoic acid (129). Furthermore, mutations in genes related to retinoic acid metabolism like RDH5 have been found to be associated with refractive errors and myopia (111, 130, 131). Deficiency of RDH5 also results in upregulation of MMP-2 and TGF-β2, promoting the epithelial-mesenchymal transition of RPE cells and myopia development (132). These findings are consistent with our observation of reduced expression levels of RDH10 in RPE exosomes of myopic eyes, indicating their role in extracellular matrix remodeling in myopia. We found that the downregulation of these RPE exosomal proteins inhibited signaling pathways related to lens development, eyes and sensory system development, visual perception, and sensory perception of the light stimulus and was associated with processes related to purine, nucleotide/nucleoside catabolism, and carboxylic acid biosynthesis, suggesting possible metabolic alterations in myopia.

### Candidate RPE exosomal signaling pathways and protein biomarkers in myopia

A comparison of our proteomics data set with published literature and the String database (63) revealed two potential exosomal protein biomarkers that were downregulated in myopic eyes: ATP6V1B2 and CRYBB2. The former has been associated with early-onset high myopia (133), while the latter has been associated with induced myopia (72). As stated previously, lower levels of CRYBB2 could be due to the oxidative stress environment of myopia eyes, enhanced by the downregulation of antioxidant genes (67) by TGF-β signaling (68). Another potential biomarker is phosphatidylethanolamine binding protein 1 (PEBP1), a Raf kinase inhibitor protein, which plays a role in cell cycle, growth, and proliferation (148). PEPB1 has been previously reported in exosomes released by the apical side of the RPE in normal physiological conditions (33) and was significantly downregulated in our myopic samples. Apart from these proteins, several other downregulated proteins in our dataset were consistently associated with myopia in the literature, providing an indirect validation of the proteomic analysis of myopic eyes (63, 76). These proteins were MYOC, ATP6V1A, RHOA, SAG, GNAT2, GNB3, COL12A1 and TGFBI.

The ingenuity pathway analysis illustrated that the differentially expressed RPE exosomal proteins of myopic eyes were involved in pathways related to phototransduction, neurotransmitters, and signal transduction, all of which are implicated in the emmetropization mechanism (15). The top canonical signaling pathways relevant to RPE exosomal proteins in myopic eyes were integrin signaling, RHOA, and cell junction pathways. Although protein RHOA was reported to be upregulated in the sclera of myopic eyes (134), it was not differentially expressed in RPE exosomes of myopic eyes in this study. Since activation of integrins and cell-cell junctions promote cell cycle progression and cell proliferation (135), these pathways likely produce changes in extracellular matrix remodeling leading to myopia (77, 136).

### *Ex-vivo* ocular assay: an innovative method with significant implications for ocular biology research

A recent comprehensive report on the causes, prevention, and treatment of myopia, released by The National Academies of Sciences, Engineering, and Medicine, highlighted the need for the development of *in vitro* experimental models that can accelerate understanding of the mechanisms of emmetropization and myopia as well as identification of candidate messengers involved in the retinoscleral signaling process (17). By successfully implementing an innovative *ex vivo* assay to explore the pathogenesis of myopia, the present study has established a model that is as simple as an *in vitro* assay but with the potential to yield more robust and physiologically relevant findings. This *ex vivo* assay model should facilitate high-impact studies of ocular mechanisms (for example, pharmacological manipulation experiments) and significantly aid in advancing the understanding of and future discoveries in ocular biology (35).

This study has several limitations, including small sample size, two different experimental myopia paradigms, and few control eyes that had recovered from prior treatments. In addition, while the neurosensory retina was carefully separated from the RPE to isolate RPE-derived exosomes, it is likely that exosomes from non-RPE cells, such as photoreceptors, were also enriched in the conditioning media. Consequently, the enriched sample was likely composed primarily, but not exclusively, of RPE exosomes. Nevertheless, the findings provide original evidence for the potential role of RPE exosomes in myopiagenesis, opening up a new avenue for understanding the molecular mechanisms behind emmetropization and myopia. Further targeted experiments are necessary to validate candidate RPE exosomal protein biomarkers and pathways identified in this study and test these proteomic signatures across different growth-modulatory conditions (e.g., induced myopia vs. recovery from myopia) and experimental models (e.g., form-deprivation, lens-induced, and limited-bandwidth). Ultimately, a thorough proteomic characterization of RPE exosomes in myopia could provide key insights into the molecular mechanism of RPE exosome-mediated growth signal transmission in the emmetropization pathway.

## Data Availability

The original contributions presented in the study are publicly available. The global mass spectrometry proteomics data presented in this study have been deposited to the ProteomeXchange Consortium via the PRIDE partner repository, accession number PXD062092.
