# Supplementary material for: Proteomic signatures of retinal pigment epithelium-derived exosomes in myopic and non-myopic tree shrew eyes
Source: Front Med (Lausanne). 2025 Apr 22;12:1523211. doi: 10.3389/fmed.2025.1523211 (PMC12052888; doi:10.3389/fmed.2025.1523211)
Supplement: Supplementary file 1 [file Data_Sheet_1.docx]

Supplementary Material

**Supplementary Figures**

*Figure S1. Functional enrichment analyses of top 41 differentially expressed RPE exosomal proteins in myopic eyes performed with ShinyGO 0.77 online tool. Enrichment of A) KEGG pathways, B) GO cellular components, and C) GO molecular functions of 21 significantly upregulated proteins in myopic samples. D) Enrichment of GO molecular functions in 17 significant downregulated proteins and 3 undetected proteins in myopic samples.*

*Figure S2. Profile of RPE exosomal proteomics compared with published data. A) Venn diagrams showing significantly downregulated RPE exosomal proteins in myopic samples (blue), significantly upregulated RPE exosomal proteins in myopic samples (red), and all identified RPE exosomal proteins (green) in this study compared with all high myopia proteins listed on STRING database (yellow). B) Same groups as in A) except that the proteins in the yellow circle are secreted from the apical side of the RPE as reported previously* (1)*.*

**
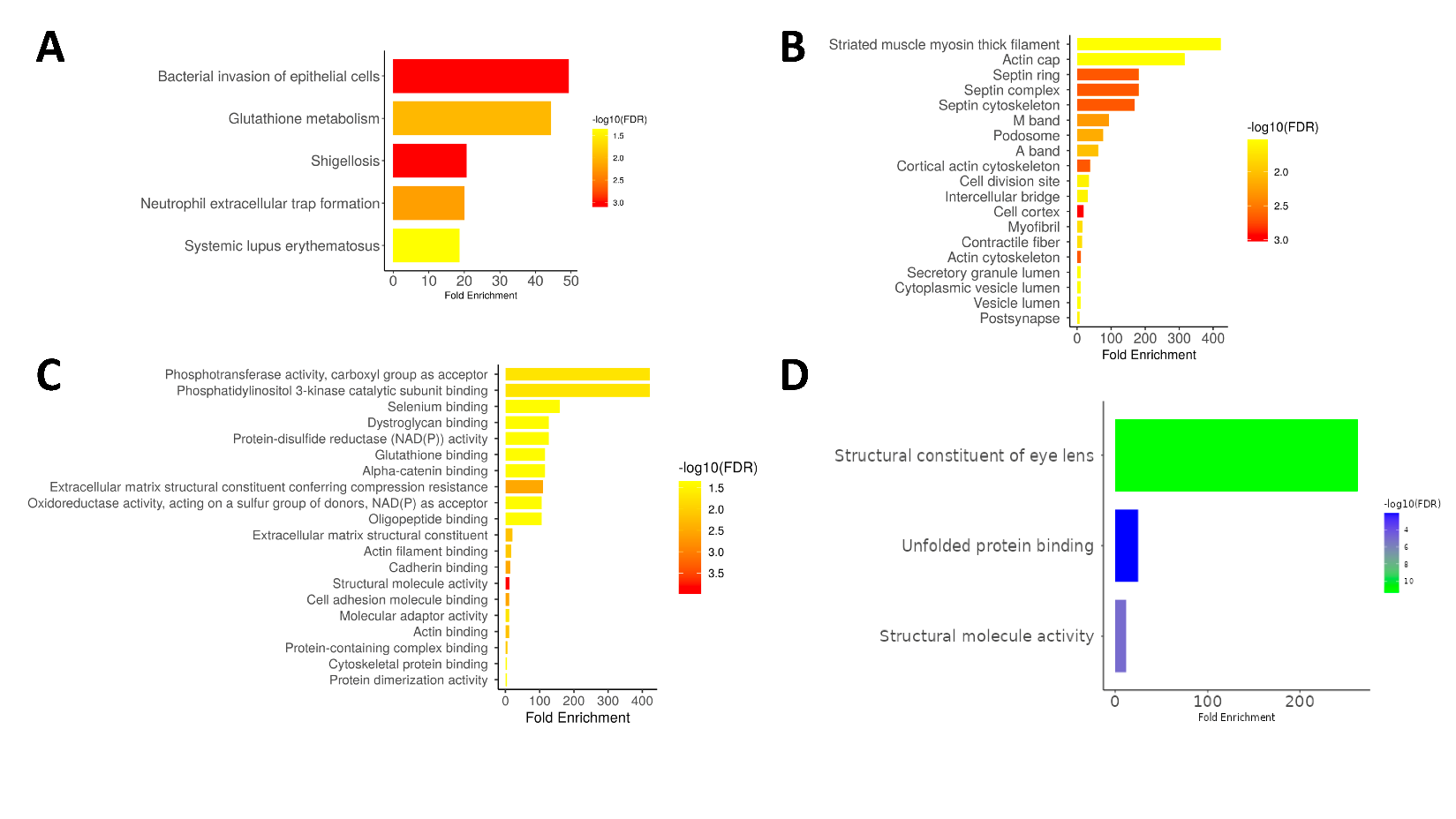
**

*Figure S1. Functional enrichment analyses of top 41 differentially expressed RPE exosomal proteins in myopic eyes performed with ShinyGO 0.77 online tool. Enrichment of A) KEGG pathways, B) GO cellular components, and C) GO molecular functions of 21 significantly upregulated proteins in myopic samples. D) Enrichment of GO molecular functions in 17 significant downregulated proteins and 3 undetected proteins in myopic samples.*

**
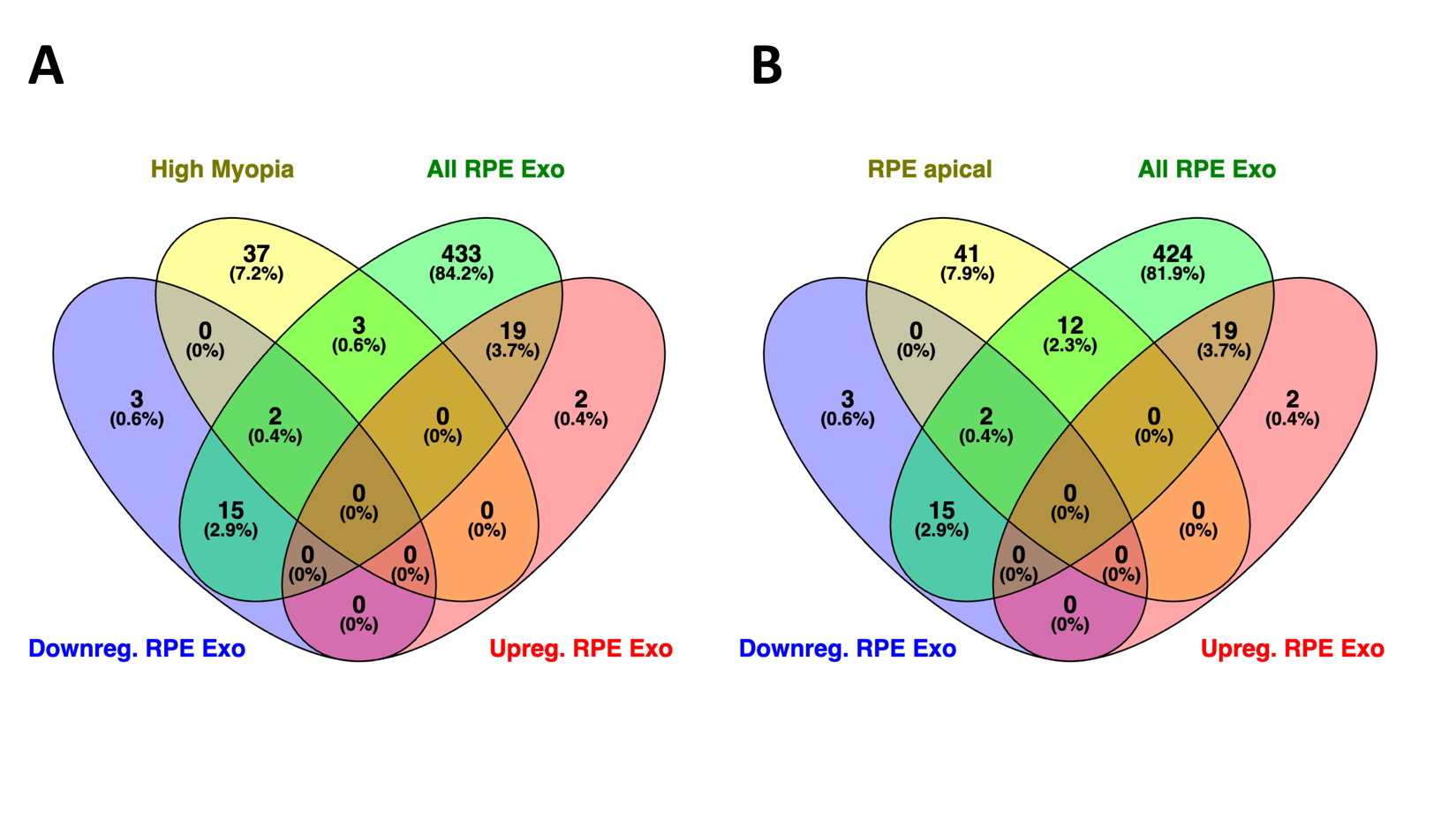
**

*Figure S2. Profile of RPE exosomal proteomics compared with published data. A) Venn diagrams showing significantly downregulated RPE exosomal proteins in myopic samples (blue), significantly upregulated RPE exosomal proteins in myopic samples (red), and all identified RPE exosomal proteins (green) in this study compared with all high myopia proteins listed on STRING database (yellow). B) Same groups as in A) except that the proteins in the yellow circle are secreted from the apical side of the RPE as reported previously* (1)*.*

**Supplementary Tables**

*Table S1. List of 48 uniquely expressed RPE exosomal proteins in myopic eyes.*

*Table S2. Biological processes of 48 uniquely expressed RPE exosomal proteins in myopic eyes.*

*Table S3. List of 41 uniquely expressed RPE exosomal proteins in non-myopic eyes.*

*Table S4. Biological processes of 41 uniquely expressed RPE exosomal proteins in non-myopic eyes.*

*Table S5. Upregulated RPE exosomal proteins in myopic eyes: Fold change, FC >1.2.*

*Table S6. Downregulated RPE exosomal proteins in myopic eyes: Fold change, FC <-1.2.*

*Table S7: IPA Diseases and biomarkers for top 38 differentially expressed proteins in myopic samples: Number of Molecules > 6.*

*Table S8. IPA Top Canonical Pathways for top 38 differentially expressed RPE exosomal proteins in myopic samples.*

*Table S9. Potential RPE exosomal protein biomarkers for myopia based on STRING database, published data* (1,2)*, and current study.*

*Table S1. List of 48 uniquely expressed RPE exosomal proteins in myopic eyes.*

| **SN** | **Identified Proteins** | **Taxonomy** | **Gene name** | **Acc#** |
| --- | --- | --- | --- | --- |
| 1 | 60S ribosomal protein L5 | Catarrhini | RL5 | P46777 |
| 2 | Adenylosuccinate synthetase isozyme 1 | Homo sapiens | PURA1 | Q8N142 |
| 3 | Adenylyl cyclase-associated protein 1 | Homo sapiens | CAP1 | Q01518 |
| 4 | Alcohol dehydrogenase class 4 mu/sigma chain | Homo sapiens | ADH7 | P40394 |
| 5 | Alpha/beta hydrolase domain-containing protein 14B | Homo sapiens | ABHEB | Q96IU4 |
| 6 | Annexin A6 | Homo sapiens | ANXA6 | P08133 |
| 7 | Apolipoprotein A-IV | Homo sapiens | APOA4 | P06727 |
| 8 | Apolipoprotein B (Including Ag(X) antigen) | Homo sapiens | C0JYY2 | C0JYY2 (+1) |
| 9 | ATP-dependent RNA helicase DDX3X | Boreoeutheria | DDX3X | O00571 |
| 10 | Ceruloplasmin | Homo sapiens | CERU | P00450 |
| 11 | Coagulation factor XIII A chain | Homo sapiens | F13A | P00488 |
| 12 | Coatomer subunit delta | Homininae | COPD | P48444 |
| 13 | Coatomer subunit gamma-1 | Catarrhini | COPG1 | Q9Y678 |
| 14 | Cochlin | Homininae | COCH | O43405 |
| 15 | Collagen alpha-3(VI) chain | Homo sapiens | CO6A3 | P12111 |
| 16 | Dermcidin | Euarchontoglires | DCD | P81605 |
| 17 | Desmin | Homininae | DESM | P17661 |
| 18 | Dolichyl-diphosphooligosaccharide--protein glycosyltransferase subunit 1 | Homo sapiens | RPN1 | P04843 |
| 19 | Ferritin | Homo sapiens | Q8TD27 | Q8TD27 |
| 20 | Filamin-C | Homo sapiens | FLNC | Q14315 |
| 21 | Fructose-1,6-bisphosphatase isozyme 2 | Homo sapiens | F16P2 | O00757 |
| 22 | Glycogen debranching enzyme | Homo sapiens | GDE | P35573 |
| 23 | Heat shock protein beta-1 | Catarrhini | HSPB1 | P04792 |
| 24 | Ig kappa chain V-II region TEW | Homo sapiens | KV204 | P01617 |
| 25 | Importin-5 | Homo sapiens | IPO5 | O00410 |
| 26 | IQ motif containing GTPase activating protein 1 | Homo sapiens | A4QPB0 | A4QPB0 (+1) |
| 27 | Kelch-like protein 41 | Homininae | KLH41 | O60662 |
| 28 | Keratocan | Homininae | KERA | O60938 |
| 29 | Kininogen 1, isoform CRA_b | Homo sapiens | B4E1C2 | B4E1C2 (+1) |
| 30 | Lambda-chain (AA -20 to 215) | Homo sapiens | A2NUT2 | A2NUT2 |
| 31 | Leukotriene A-4 hydrolase | Homininae | LKHA4 | P09960 |
| 32 | Liver histone H1e | Homo sapiens | A3R0T7 | A3R0T7 (+2) |
| 33 | Myocilin | Homo sapiens | MYOC | Q99972 |
| 34 | Myosin-binding protein C, fast-type | Homo sapiens | MYPC2 | Q14324 |
| 35 | Phosducin | Homo sapiens | PHOS | P20941 |
| 36 | Phosphoacetylglucosamine mutase | Homo sapiens | AGM1 | O95394 |
| 37 | Phosphoglucomutase-2 | Homo sapiens | PGM2 | Q96G03 |
| 38 | Phosphoglucomutase-like protein 5 | Hominidae | PGM5 | Q15124 |
| 39 | Proteasome subunit alpha type-5 | Eutheria | PSA5 | P28066 |
| 40 | Serine/threonine-protein phosphatase 2A activator | Homo sapiens | PTPA | Q15257 |
| 41 | Serine/threonine-protein phosphatase PP1-beta catalytic subunit | Amniota | PP1B | P62140 |
| 42 | Sorting nexin-2 | Homininae | SNX2 | O60749 |
| 43 | Staphylococcal nuclease domain-containing protein 1 | Homo sapiens | SND1 | Q7KZF4 |
| 44 | Thrombospondin-1 | Homo sapiens | TSP1 | P07996 |
| 45 | Transforming growth factor-beta-induced protein ig-h3 | Homininae | BGH3 | Q15582 |
| 46 | Tropomyosin 1 (Alpha) isoform 4 | Homininae | D9YZV5 | D9YZV5 |

*Table S2. Biological processes of 48 uniquely expressed RPE exosomal proteins in myopic eyes.*

| **N** | **High level GO category** | **Gene name: Unique proteins** |
| --- | --- | --- |
| 11 | GO:0009653 anatomical structure morphogenesis | CAP1 THBS1 PGM5 ANXA6 MYOC SNX2 KLHL41 TGFBI COCH HSPB1 FLNC |
| 11 | GO:0065008 regulation of biological quality | HSPB1 CP APOA4 F13A1 ADH7 ANXA6 MYOC THBS1 COCH RPL5 DDX3X |
| 11 | GO:0065009 regulation of molecular function | APOA4 CAP1 PTPA RPL5 THBS1 DDX3X HSPB1 PDC COL6A3 ADH7 IPO5 |
| 10 | GO:0003008 system process | APOA4 ANXA6 MYOC MYBPC2 COCH PDC TGFBI KERA DES KLHL41 |
| 10 | GO:0009605 response to external stimulus | APOA4 THBS1 DCD ADH7 DDX3X COCH AGL PPP1CB HSPB1 PDC |
| 10 | GO:0051234 establishment of localization | CP IPO5 ARCN1 APOA4 COPG1 SNX2 THBS1 CAP1 ANXA6 HSPB1 |
| 9 | GO:0007155 cell adhesion | HSPB1 TGFBI MYOC APOA4 THBS1 PPP1CB MYBPC2 PGM5 COL6A3 |
| 9 | GO:0009056 catabolic process | APOA4 LTA4H PSMA5 AGL SND1 RPL5 PGM2 ADH7 HSPB1 |
| 8 | GO:0006950 response to stress | HSPB1 F13A1 ANXA6 APOA4 THBS1 DCD DDX3X COCH |
| 8 | GO:0033036 macromolecule localization | IPO5 APOA4 COPG1 THBS1 ARCN1 SNX2 HSPB1 DDX3X |
| 8 | GO:0042221 response to chemical | APOA4 THBS1 ADH7 DDX3X IPO5 HSPB1 LTA4H AGL |
| 8 | GO:0044085 cellular component biogenesis | APOA4 RPL5 PGM5 MYOC DDX3X KLHL41 FLNC THBS1 |
| 8 | GO:0048583 regulation of response to stimulus | MYOC THBS1 DDX3X COCH HSPB1 PDC ADH7 RPL5 |
| 7 | GO:0009893 positive regulation of metabolic process | APOA4 ABHD14B PTPA RPL5 THBS1 DDX3X HSPB1 |
| 7 | GO:0023051 regulation of signaling | MYOC THBS1 DDX3X HSPB1 PDC ADH7 RPL5 |
| 7 | GO:0051641 cellular localization | IPO5 ARCN1 COPG1 ANXA6 SNX2 HSPB1 DDX3X |
| 6 | GO:0002376 immune system process | PGM3 APOA4 THBS1 DDX3X COCH ADSS1 |
| 6 | GO:0009607 response to biotic stimulus | APOA4 DCD ADH7 DDX3X COCH HSPB1 |
| 6 | GO:0032879 regulation of localization | APOA4 ANXA6 MYOC THBS1 IPO5 HSPB1 |
| 6 | GO:0044419 biological process involved in interspecies interaction between organisms | APOA4 DCD ADH7 DDX3X COCH HSPB1 |
| 6 | GO:0048646 anatomical structure formation involved in morphogenesis | THBS1 PGM5 KLHL41 TGFBI HSPB1 FLNC |
| 6 | GO:0051239 regulation of multicellular organismal process | APOA4 THBS1 ANXA6 DDX3X HSPB1 DES |
| 6 | GO:0051707 response to other organism | APOA4 DCD ADH7 DDX3X COCH HSPB1 |
| 5 | GO:0040011 locomotion | ANXA6 MYOC THBS1 CAP1 HSPB1 |
| 5 | GO:0009628 response to abiotic stimulus | HSPB1 DDX3X THBS1 PPP1CB PDC |
| 5 | GO:0048870 cell motility | ANXA6 MYOC THBS1 CAP1 HSPB1 |
| 5 | GO:0050793 regulation of developmental process | THBS1 KLHL41 MYOC COCH HSPB1 |
| 5 | GO:0051674 localization of cell | ANXA6 MYOC THBS1 CAP1 HSPB1 |
| 4 | GO:0006955 immune response | APOA4 DDX3X COCH THBS1 |
| 4 | GO:0009719 response to endogenous stimulus | THBS1 IPO5 LTA4H AGL |
| 4 | GO:0023057 negative regulation of signaling | MYOC THBS1 HSPB1 DDX3X |
| 4 | GO:0030155 regulation of cell adhesion | MYOC THBS1 PPP1CB TGFBI |
| 3 | GO:0002682 regulation of immune system process | THBS1 DDX3X COCH |
| 3 | GO:0008283 cell population proliferation | KLHL41 THBS1 TGFBI |
| 3 | GO:0022402 cell cycle process | PTPA SND1 DDX3X |
| 3 | GO:0040012 regulation of locomotion | MYOC THBS1 HSPB1 |
| 3 | GO:0051094 positive regulation of developmental process | MYOC HSPB1 THBS1 |
| 3 | GO:0051240 positive regulation of multicellular organismal process | THBS1 DDX3X HSPB1 |
| 2 | GO:0000003 reproduction | DDX3X PGM3 |
| 2 | GO:0007610 behavior | ARCN1 THBS1 |
| 2 | GO:0022414 reproductive process | DDX3X PGM3 |
| 2 | GO:0040007 growth | ANXA6 DDX3X |
| 2 | GO:0002684 positive regulation of immune system process | THBS1 COCH |
| 2 | GO:0019953 sexual reproduction | DDX3X PGM3 |
| 2 | GO:0032504 multicellular organism reproduction | DDX3X PGM3 |
| 2 | GO:0042330 taxis | THBS1 HSPB1 |
| 2 | GO:0048609 multicellular organismal reproductive process | DDX3X PGM3 |
| 1 | GO:0001906 cell killing | DCD |
| 1 | GO:0048511 rhythmic process | PPP1CB |
| 1 | GO:0002520 immune system development | PGM3 |
| 1 | GO:0002683 negative regulation of immune system process | THBS1 |
| 1 | GO:0003006 developmental process involved in reproduction | PGM3 |
| 1 | GO:0006457 protein folding | HSPB1 |
| 1 | GO:0006914 autophagy | HSPB1 |
| 1 | GO:0007623 circadian rhythm | PPP1CB |
| 1 | GO:0007626 locomotory behavior | ARCN1 |
| 1 | GO:0016049 cell growth | DDX3X |
| 1 | GO:0019882 antigen processing and presentation | THBS1 |
| 1 | GO:0031640 killing of cells of another organism | DCD |
| 1 | GO:0040008 regulation of growth | DDX3X |
| 1 | GO:0040013 negative regulation of locomotion | THBS1 |
| 1 | GO:0042445 hormone metabolic process | ADH7 |
| 1 | GO:0042752 regulation of circadian rhythm | PPP1CB |
| 1 | GO:0043473 pigmentation | ARCN1 |
| 1 | GO:0043500 muscle adaptation | MYOC |
| 1 | GO:0045321 leukocyte activation | THBS1 |
| 1 | GO:0048589 developmental growth | ANXA6 |
| 1 | GO:0050900 leukocyte migration | THBS1 |
| 1 | GO:0051093 negative regulation of developmental process | THBS1 |
| 1 | GO:0051235 maintenance of location | ANXA6 |
| 1 | GO:0051606 detection of stimulus | PDC |
| 1 | GO:0072376 protein activation cascade | F13A1 |
| 1 | GO:0097006 regulation of plasma lipoprotein particle levels | APOA4 |

*Table S3. List of 41 uniquely expressed RPE exosomal proteins in non-myopic eyes*

| **SN** | **Identified Proteins** | **Taxonomy** | **Gene name** | **Acc#** |
| --- | --- | --- | --- | --- |
| 1 | 40S ribosomal protein S16 | Theria | RS16 | P62249 |
| 2 | 40S ribosomal protein S20 | Boreoeutheria | RS20 | P60866 |
| 3 | Beta-arrestin-2 | Hominoidea | ARRB2 | P32121 |
| 4 | Beta-crystallin A2 | Homo sapiens | CRBA2 | P53672 |
| 5 | Beta-crystallin A3 | Homo sapiens | CRBA1 | P05813 |
| 6 | Beta-crystallin B1 | Homo sapiens | CRBB1 | P53674 |
| 7 | Beta-crystallin B3 | Homo sapiens | CRBB3 | P26998 |
| 8 | CDP-diacylglycerol--inositol 3-phosphatidyltransferase | Homininae | CDIPT | O14735 |
| 9 | Clathrin light chain A | Catarrhini | CLCA | P09496 |
| 10 | Cone cGMP-specific 3',5'-cyclic phosphodiesterase subunit alpha' | Homo sapiens | PDE6C | P51160 |
| 11 | Coronin-1B | Homo sapiens | COR1B | Q9BR76 |
| 12 | Dihydrolipoyl dehydrogenase, mitochondrial | Homininae | DLDH | P09622 |
| 13 | Epididymis secretory sperm binding protein Li 124m | Homo sapiens | V9HW84 | V9HW84 |
| 14 | Eukaryotic translation initiation factor 2 subunit 1 | Catarrhini | IF2A | P05198 |
| 15 | Fumarate hydratase, mitochondrial | Homo sapiens | FUMH | P07954 |
| 16 | Gamma-crystallin C | Homo sapiens | CRGC | P07315 |
| 17 | Keratin, type I cytoskeletal 17 | Homo sapiens | K1C17 | Q04695 |
| 18 | Ketimine reductase mu-crystallin | Homininae | CRYM | Q14894 |
| 19 | Malectin | Homininae | MLEC | Q14165 |
| 20 | Myosin regulatory light chain 12B | Theria | ML12B | O14950 (+1) |
| 21 | N-alpha-acetyltransferase 35, NatC auxiliary subunit | Hominidae | NAA35 | Q5VZE5 |
| 22 | Na(+)/H(+) exchange regulatory cofactor NHE-RF1 | Homo sapiens | NHRF1 | O14745 |
| 23 | Phosphate carrier protein, mitochondrial | Homininae | MPCP | Q00325 |
| 24 | Plastin-3 | Simiiformes | PLST | P13797 |
| 25 | PRA1 family protein 2 | Homininae | PRAF2 | O60831 |
| 26 | Protein canopy homolog 2 | Homininae | CNPY2 | Q9Y2B0 |
| 27 | Protein S100-A4 | Catarrhini | S10A4 | P26447 |
| 28 | Pyruvate dehydrogenase E1 component subunit alpha, somatic form, mitochondrial | Simiiformes | ODPA | P08559 |
| 29 | Pyruvate dehydrogenase E1 component subunit beta, mitochondrial | Catarrhini | ODPB | P11177 |
| 30 | Ras-related protein Rab-18 | Simiiformes | RAB18 | Q9NP72 |
| 31 | Retinoschisin | Catarrhini | XLRS1 | O15537 |
| 32 | Serine/threonine-protein kinase SMG1 | Homo sapiens | SMG1 | Q96Q15 |
| 33 | Succinyl-CoA ligase [ADP-forming] subunit beta, mitochondrial | Homo sapiens | SUCB1 | Q9P2R7 |
| 34 | T-complex protein 1 subunit beta | Catarrhini | TCPB | P78371 |
| 35 | T-complex protein 1 subunit delta | Simiiformes | TCPD | P50991 |
| 36 | T-complex protein 1 subunit eta | Homininae | TCPH | Q99832 |
| 37 | Transmembrane emp24 domain-containing protein 9 | Homo sapiens | TMED9 | Q9BVK6 |
| 38 | tRNA-splicing ligase RtcB homolog | Boreoeutheria | RTCB | Q9Y3I0 |
| 39 | Ubiquitin carboxyl-terminal hydrolase 5 | Homininae | UBP5 | P45974 |
| 40 | V-type proton ATPase subunit H | Hominoidea | VATH | Q9UI12 |
| 41 | Vitamin D-binding protein | Homo sapiens | VTDB | P02774 |

*Table S4. Biological processes of 41 uniquely expressed RPE exosomal proteins in non-myopic eyes.*

| **N** | **High level GO category** | **Genes name : Unique proteins** |
| --- | --- | --- |
| 15 | GO:0051234 establishment of localization | SLC25A3 RAB18 CLTA ARRB2 TMED9 ATP6V1H CRYBA1 SLC9A3R1 CCT4 CCT7 GC CCT2 PRAF2 CRYM SMG1 |
| 11 | GO:0033036 macromolecule localization | CCT4 CCT7 CCT2 RAB18 SLC9A3R1 TMED9 CORO1B CLTA ARRB2 PRAF2 SMG1 |
| 10 | GO:0065008 regulation of biological quality | CRYM ATP6V1H DLD FH SLC9A3R1 MYL12B CCT4 CCT7 CCT2 ARRB2 |
| 9 | GO:0003008 system process | PDE6C CRYBB3 CRYBB1 CRYBA1 CRYBA2 RS1 SLC9A3R1 ARRB2 CRYM |
| 9 | GO:0051641 cellular localization | CCT4 CCT7 CCT2 RAB18 SLC9A3R1 TMED9 CORO1B CLTA SMG1 |
| 8 | GO:0009056 catabolic process | SMG1 SUCLA2 DLD CRYBA1 USP5 ARRB2 ATP6V1H CRYM |
| 8 | GO:0044085 cellular component biogenesis | PLS3 RPS16 CLTA RS1 CORO1B EIF2S1 SLC9A3R1 CCT2 |
| 8 | GO:0048583 regulation of response to stimulus | ARRB2 S100A4 RPS20 SLC9A3R1 SMG1 RS1 CRYBA1 CORO1B |
| 8 | GO:0065009 regulation of molecular function | ARRB2 RPS20 ATP6V1H SLC9A3R1 EIF2S1 CNPY2 CCT4 CCT2 |
| 7 | GO:0009653 anatomical structure morphogenesis | CORO1B DLD PDE6C RS1 SLC9A3R1 MYL12B KRT17 |
| 7 | GO:0009893 positive regulation of metabolic process | FH KRT17 ARRB2 USP5 CCT4 CCT7 CCT2 |
| 6 | GO:0000003 reproduction | DLD RTCB CCT4 CCT7 ARRB2 CCT2 |
| 6 | GO:0022414 reproductive process | DLD RTCB CCT4 CCT7 ARRB2 CCT2 |
| 6 | GO:0023051 regulation of signaling | ARRB2 S100A4 RPS20 SLC9A3R1 RS1 CRYBA1 |
| 6 | GO:0032879 regulation of localization | CCT4 CCT7 CCT2 SLC9A3R1 ARRB2 CORO1B |
| 6 | GO:0051239 regulation of multicellular organismal process | FH CRYBA1 SLC9A3R1 KRT17 ARRB2 CNPY2 |
| 5 | GO:0006950 response to stress | EIF2S1 SMG1 CORO1B FH ARRB2 |
| 5 | GO:0009605 response to external stimulus | CORO1B PDE6C RS1 EIF2S1 ARRB2 |
| 5 | GO:0042221 response to chemical | EIF2S1 ARRB2 CORO1B RPS16 SLC9A3R1 |
| 5 | GO:0050793 regulation of developmental process | CORO1B MYL12B KRT17 ARRB2 SLC9A3R1 |
| 4 | GO:0009628 response to abiotic stimulus | PDE6C RS1 EIF2S1 ARRB2 |
| 4 | GO:0019953 sexual reproduction | DLD CCT4 CCT7 CCT2 |
| 3 | GO:0040011 locomotion | CORO1B SLC9A3R1 ARRB2 |
| 3 | GO:0003006 developmental process involved in reproduction | DLD RTCB ARRB2 |
| 3 | GO:0006457 protein folding | CCT4 CCT7 CCT2 |
| 3 | GO:0006914 autophagy | SMG1 CRYBA1 ATP6V1H |
| 3 | GO:0009719 response to endogenous stimulus | ARRB2 SLC9A3R1 CORO1B |
| 3 | GO:0023057 negative regulation of signaling | ARRB2 SLC9A3R1 CRYBA1 |
| 3 | GO:0048870 cell motility | CORO1B SLC9A3R1 ARRB2 |
| 3 | GO:0051094 positive regulation of developmental process | CORO1B KRT17 ARRB2 |
| 3 | GO:0051240 positive regulation of multicellular organismal process | FH KRT17 ARRB2 |
| 3 | GO:0051606 detection of stimulus | PDE6C RS1 ARRB2 |
| 3 | GO:0051674 localization of cell | CORO1B SLC9A3R1 ARRB2 |
| 2 | GO:0008283 cell population proliferation | SLC9A3R1 NAA35 |
| 2 | GO:0032504 multicellular organism reproduction | DLD ARRB2 |
| 2 | GO:0040012 regulation of locomotion | SLC9A3R1 CORO1B |
| 2 | GO:0040013 negative regulation of locomotion | SLC9A3R1 CORO1B |
| 2 | GO:0042330 taxis | CORO1B ARRB2 |
| 2 | GO:0048609 multicellular organismal reproductive process | DLD ARRB2 |
| 1 | GO:0001906 cell killing | ARRB2 |
| 1 | GO:0002376 immune system process | ARRB2 |
| 1 | GO:0007610 behavior | ARRB2 |
| 1 | GO:0040007 growth | KRT17 |
| 1 | GO:0048511 rhythmic process | ARRB2 |
| 1 | GO:0001909 leukocyte mediated cytotoxicity | ARRB2 |
| 1 | GO:0002252 immune effector process | ARRB2 |
| 1 | GO:0002682 regulation of immune system process | ARRB2 |
| 1 | GO:0002683 negative regulation of immune system process | ARRB2 |
| 1 | GO:0006955 immune response | ARRB2 |
| 1 | GO:0007155 cell adhesion | RS1 |
| 1 | GO:0007626 locomotory behavior | ARRB2 |
| 1 | GO:0009607 response to biotic stimulus | ARRB2 |
| 1 | GO:0016049 cell growth | KRT17 |
| 1 | GO:0031341 regulation of cell killing | ARRB2 |
| 1 | GO:0040008 regulation of growth | KRT17 |
| 1 | GO:0042445 hormone metabolic process | CRYM |
| 1 | GO:0044419 biological process involved in interspecies interaction between organisms | ARRB2 |
| 1 | GO:0048646 anatomical structure formation involved in morphogenesis | RS1 |
| 1 | GO:0051707 response to other organism | ARRB2 |
| 1 | GO:0097006 regulation of plasma lipoprotein particle levels | CNPY2 |
| 1 | GO:1905097 regulation of guanyl-nucleotide exchange factor activity | EIF2S1 |

*Table S5. Upregulated RPE exosomal proteins in myopic eyes: Fold change, FC >1.2.*

| **SN** | **UniProtKB Names** | **Acc#** | **SAM** | **T-test** | **Fold Change (Myopic/**  **Non-myopic)** |
| --- | --- | --- | --- | --- | --- |
| 1 | Histone H4 | P62805 | 0.69 | 0.10 | 3.04 |
| 2 | EGF-containing fibulin-like extracellular matrix protein 1 | Q12805 | 0.59 | 0.14 | 2.96 |
| 3 | Alpha-actinin-2 | P35609 | 0.36 | 0.23 | 2.76 |
| 4 | Histone H2A.J | Q9BTM1 | 0.54 | 0.11 | 2.75 |
| 5 | PTB domain-containing engulfment adapter protein 1 | Q9UBP9 | 1.04 | 0.08 | 2.59 |
| 6 | Histone H3.1 | P68431 | 0.63 | 0.14 | 2.59 |
| 7 | AP-2 complex subunit alpha-1 | O95782 | 0.86 | 0.11 | 2.43 |
| 8 | Aldehyde dehydrogenase, dimeric NADP-preferring | P30838 | 0.40 | 0.20 | 2.40 |
| 9 | Lumican | P51884 | 1.44 | 0.01 | 2.27 |
| 10 | Vinculin | P18206 | 0.69 | 0.09 | 2.22 |
| 11 | Glutathione peroxidase 3 | P22352 | 0.92 | 0.06 | 2.12 |
| 12 | Spectrin beta chain, non-erythrocytic 1 | Q01082 | 0.73 | 0.03 | 1.94 |
| 13 | Septin-7 | Q16181 | 0.88 | 0.01 | 1.91 |
| 14 | 60S ribosomal protein L10a | P62906 | 0.58 | 0.09 | 1.84 |
| 15 | Protein DJ-1 | K7ELW0 | 0.40 | 0.21 | 1.83 |
| 16 | BTB/POZ domain-containing protein KCTD12 | Q96CX2 | 0.71 | 0.07 | 1.82 |
| 17 | Fibrinogen gamma chain | P02679 | 0.83 | 0.01 | 1.80 |
| 18 | Osteoglycin OG | Q7Z532 | 0.50 | 0.09 | 1.79 |
| 19 | Fibromodulin | Q06828 | 0.58 | 0.10 | 1.73 |
| 20 | FN1 protein | B7ZLE5 | 0.36 | 0.16 | 1.72 |
| 21 | Filamin-A | P21333 | 0.41 | 0.14 | 1.72 |
| 22 | Phosphoglycerate kinase 1 | P00558 | 0.68 | 0.03 | 1.70 |
| 23 | Septin-2 | Q15019 | 0.78 | 0.04 | 1.70 |
| 24 | Alpha-actinin-4 | O43707 | 0.49 | 0.10 | 1.65 |
| 25 | Glycogen phosphorylase, muscle form | P11217 | 0.21 | 0.31 | 1.65 |
| 26 | Glutathione S-transferase Mu 3 | P21266 | 0.88 | 0.02 | 1.64 |
| 27 | Protein disulfide-isomerase | P07237 | 0.22 | 0.33 | 1.61 |
| 28 | Gelsolin | P06396 | 0.79 | 0.03 | 1.57 |
| 29 | Fibrinogen beta chain | P02675 | 0.35 | 0.23 | 1.55 |
| 30 | Extracellular superoxide dismutase [Cu-Zn] | P08294 | 0.51 | 0.16 | 1.50 |
| 31 | Myomesin-1 | P52179 | 0.92 | 0.01 | 1.50 |
| 32 | Elongation factor 2 | P13639 | 0.41 | 0.12 | 1.50 |
| 33 | Serum albumin | P02769 | 0.20 | 0.29 | 1.49 |
| 34 | Ras-related protein Rap-1b | P61224 | 0.42 | 0.11 | 1.47 |
| 35 | Thrombospondin-4 | P35443 | 0.40 | 0.11 | 1.47 |
| 36 | Annexin A1 | P04083 | 0.36 | 0.21 | 1.46 |
| 37 | 40S ribosomal protein SA | P08865 | 0.68 | 0.05 | 1.45 |
| 38 | Heterogeneous nuclear ribonucleoprotein H3 | P31942 | 0.19 | 0.33 | 1.44 |
| 39 | Cofilin-1 | P23528 | 0.23 | 0.27 | 1.39 |
| 40 | Complement C3 | P01024 | 0.32 | 0.18 | 1.38 |
| 41 | Peroxiredoxin-6 | P30041 | 0.30 | 0.29 | 1.38 |
| 42 | EH domain-containing protein 1 | Q9H4M9 | 0.27 | 0.24 | 1.38 |
| 43 | Transketolase | P29401 | 0.46 | 0.11 | 1.38 |
| 44 | 4F2 cell-surface antigen heavy chain | P08195 | 0.26 | 0.27 | 1.37 |
| 45 | Phosphoglycerate mutase 2 | P15259 | 0.80 | 0.03 | 1.37 |
| 46 | Adenosylhomocysteinase | P23526 | 0.23 | 0.28 | 1.36 |
| 47 | Heat shock 70 kDa protein 12A | O43301 | 0.22 | 0.28 | 1.36 |
| 48 | Putative adenosylhomocysteinase 3 | Q96HN2 | 0.29 | 0.27 | 1.36 |
| 49 | Rab GDP dissociation inhibitor alpha | P31150 | 0.29 | 0.24 | 1.35 |
| 50 | Procollagen C-endopeptidase enhancer 1 | Q15113 | 0.67 | 0.06 | 1.35 |
| 51 | Clusterin | P10909 | 0.42 | 0.17 | 1.34 |
| 52 | Spectrin alpha chain, non-erythrocytic 1 | Q13813 | 0.39 | 0.13 | 1.33 |
| 53 | Heterogeneous nuclear ribonucleoprotein F | P52597 | 0.42 | 0.21 | 1.33 |
| 54 | Erlin-2 | O94905 | 0.24 | 0.25 | 1.33 |
| 55 | Guanine nucleotide-binding protein subunit beta-2-like 1 | P63244 | 0.30 | 0.22 | 1.32 |
| 56 | Glutathione S-transferase Mu 5 | P46439 | 0.38 | 0.19 | 1.30 |
| 57 | Microtubule-associated protein 1B | P46821 | 0.34 | 0.24 | 1.30 |
| 58 | Actin-related protein 3 | P61158 | 0.30 | 0.20 | 1.29 |
| 59 | Rab GDP dissociation inhibitor beta | P50395 | 0.31 | 0.21 | 1.28 |
| 60 | Phosphoglucomutase-1 | P36871 | 0.26 | 0.25 | 1.28 |
| 61 | Splicing factor, proline- and glutamine-rich | P23246 | 0.23 | 0.32 | 1.28 |
| 62 | 14-3-3 protein gamma | P61981 | 0.43 | 0.10 | 1.27 |
| 63 | Destrin | P60981 | 0.16 | 0.32 | 1.27 |
| 64 | Retinol dehydrogenase 11 | Q8TC12 | 0.63 | 0.05 | 1.27 |
| 65 | Clathrin heavy chain 1 | Q00610 | 0.36 | 0.14 | 1.26 |
| 66 | 14-3-3 protein epsilon | P62258 | 0.49 | 0.07 | 1.26 |
| 67 | Transitional endoplasmic reticulum ATPase | P55072 | 0.22 | 0.26 | 1.24 |
| 68 | Pigment epithelium-derived factor | P36955 | 0.24 | 0.26 | 1.24 |
| 69 | Heterogeneous nuclear ribonucleoprotein A1 | P09651 | 0.19 | 0.31 | 1.24 |
| 70 | Ubiquitin-conjugating enzyme E2 N | P61088 | 0.39 | 0.13 | 1.22 |
| 71 | Glucose-6-phosphate isomerase | P06744 | 0.18 | 0.29 | 1.22 |
| 72 | 14-3-3 protein beta/alpha | P31946 | 0.32 | 0.16 | 1.22 |
| 73 | Actin-related protein 2/3 complex subunit 4 | P59998 | 0.31 | 0.22 | 1.22 |
| 74 | PKM2 protein | Q504U3 | 0.34 | 0.19 | 1.21 |
| 75 | Epoxide hydrolase 1 | P07099 | 0.16 | 0.31 | 1.21 |
| 76 | Heparin cofactor 2 | P05546 | 0.30 | 0.20 | 1.20 |
| 77 | P37 AUF1 | Q12771 | 0.13 | 0.35 | 1.20 |
| 78 | Heat shock 70 kDa protein 1A/1B | P08107 | 0.34 | 0.16 | 1.20 |
| 79 | Inter-alpha (Globulin) inhibitor H4 (Plasma Kallikrein-sensitive glycoprotein) | B2RMS9 | 0.28 | 0.19 | 1.20 |

*Table S6. Downregulated RPE exosomal proteins in myopic eyes: Fold change, FC <-1.2.*

| **SN** | **UniProtKB Names** | **Acc#** | **SAM** | **T-test** | **Fold Change (Myopic/**  **Non-myopic)** |
| --- | --- | --- | --- | --- | --- |
| 1 | ATP synthase subunit beta, mitochondrial | P06576 | -0.38 | 0.16 | -2.79 |
| 2 | 40S ribosomal protein S5 | P46782 | -1.25 | 0.00 | -2.41 |
| 3 | Cytosolic acyl coenzyme A thioester hydrolase | O00154 | -0.74 | 0.04 | -2.15 |
| 4 | Beta-crystallin B2 | P43320 | -0.63 | 0.05 | -2.14 |
| 5 | ADP-ribosylation factor 3 | P61204 | -0.66 | 0.07 | -2.12 |
| 6 | Poly(rC)-binding protein 2 | Q15366 | -1.17 | 0.01 | -2.10 |
| 7 | Retinol dehydrogenase 10 | Q8IZV5 | -0.49 | 0.08 | -1.86 |
| 8 | Histidine triad nucleotide-binding protein 1 | P49773 | -0.53 | 0.06 | -1.83 |
| 9 | Opsin 1 (Cone pigments), medium-wave-sensitive | B7ZLG5 | -0.53 | 0.07 | -1.81 |
| 10 | Phosphatidylethanolamine-binding protein 1 | P30086 | -0.53 | 0.07 | -1.80 |
| 11 | 6-phosphogluconate dehydrogenase, decarboxylating | P52209 | -0.63 | 0.04 | -1.79 |
| 12 | Alpha-crystallin B chain | P02511 | -0.53 | 0.07 | -1.75 |
| 13 | Long-chain-fatty-acid--CoA ligase 6 | Q9UKU0 | -0.32 | 0.18 | -1.71 |
| 14 | Beta-globin | C8C504 | -0.37 | 0.13 | -1.70 |
| 15 | Phosphoserine aminotransferase | Q9Y617 | -0.58 | 0.07 | -1.70 |
| 16 | Atlastin-3 | Q6DD88 | -0.23 | 0.25 | -1.70 |
| 17 | CDC37 protein | Q6FG59 | -0.81 | 0.04 | -1.64 |
| 18 | Alpha-crystallin A chain | P02489 | -0.64 | 0.03 | -1.61 |
| 19 | Cytochrome b5 | P00167 | -0.33 | 0.17 | -1.61 |
| 20 | V-type proton ATPase subunit B, brain isoform | P21281 | -0.45 | 0.09 | -1.59 |
| 21 | Arrestin-C | P36575 | -0.49 | 0.10 | -1.57 |
| 22 | Serum paraoxonase/lactonase 3 | Q15166 | -0.36 | 0.13 | -1.56 |
| 23 | Creatine kinase U-type, mitochondrial | P12532 | -0.40 | 0.11 | -1.55 |
| 24 | Cell growth-inhibiting protein 34 | Q08ES8 | -0.68 | 0.05 | -1.53 |
| 25 | ATP synthase subunit alpha, mitochondrial | P25705 | -0.21 | 0.26 | -1.52 |
| 26 | Malate dehydrogenase, mitochondrial | P40926 | -0.36 | 0.16 | -1.52 |
| 27 | Glucose-6-phosphate 1-dehydrogenase | P11413 | -0.24 | 0.22 | -1.48 |
| 28 | Syntaxin-binding protein 1 | P61764 | -0.66 | 0.03 | -1.47 |
| 29 | Protein ERGIC-53 | P49257 | -0.32 | 0.16 | -1.45 |
| 30 | Calretinin | P22676 | -0.26 | 0.21 | -1.43 |
| 31 | Vesicle-fusing ATPase | P46459 | -0.45 | 0.10 | -1.42 |
| 32 | Sodium/potassium-transporting ATPase subunit beta-1 | P05026 | -0.31 | 0.19 | -1.41 |
| 33 | Class IVb beta tubulin | Q8IWP6 | -0.76 | 0.03 | -1.40 |
| 34 | Guanine nucleotide-binding protein G(I)/G(S)/G(T) subunit beta-3 | P16520 | -0.44 | 0.11 | -1.39 |
| 35 | Hexokinase-1 | P19367 | -0.33 | 0.21 | -1.39 |
| 36 | Cullin-associated NEDD8-dissociated protein 1 | Q86VP6 | -0.21 | 0.26 | -1.38 |
| 37 | Dihydropyrimidinase-related protein 2 | Q16555 | -0.38 | 0.12 | -1.37 |
| 38 | Phosphatidylinositol transfer protein alpha isoform | Q00169 | -0.29 | 0.21 | -1.37 |
| 39 | Reticulon-3 | O95197 | -0.45 | 0.11 | -1.37 |
| 40 | DPYSL3 protein | Q6DEN2 | -0.55 | 0.06 | -1.35 |
| 41 | Inward rectifier potassium channel 13 | O60928 | -0.33 | 0.15 | -1.35 |
| 42 | Aspartate aminotransferase, cytoplasmic | P17174 | -0.21 | 0.26 | -1.34 |
| 43 | Alanine--tRNA ligase, cytoplasmic | P49588 | -0.25 | 0.21 | -1.33 |
| 44 | Inorganic pyrophosphatase | Q15181 | -0.26 | 0.21 | -1.32 |
| 45 | Fructose-bisphosphate aldolase C | P09972 | -0.78 | 0.02 | -1.31 |
| 46 | Peptidyl-prolyl cis-trans isomerase FKBP1A | P62942 | -0.34 | 0.17 | -1.30 |
| 47 | Sodium/potassium-transporting ATPase subunit alpha-3 | P13637 | -0.55 | 0.05 | -1.29 |
| 48 | Prenylcysteine oxidase 1 | Q9UHG3 | -0.28 | 0.19 | -1.28 |
| 49 | Keratin, type I cytoskeletal 16 | P08779 | -0.16 | 0.34 | -1.28 |
| 50 | Aspartate aminotransferase, mitochondrial | P00505 | -0.33 | 0.16 | -1.27 |
| 51 | Serum paraoxonase/arylesterase 1 | P27169 | -0.34 | 0.17 | -1.27 |
| 52 | Heterogeneous nuclear ribonucleoprotein L | P14866 | -0.31 | 0.20 | -1.27 |
| 53 | Ras-related C3 botulinum toxin substrate 1 | P63000 | -0.29 | 0.20 | -1.27 |
| 54 | Receptor expression-enhancing protein 5 | Q00765 | -0.25 | 0.22 | -1.27 |
| 55 | Tubulin beta-3 chain | Q13509 | -0.34 | 0.15 | -1.26 |
| 56 | Prolow-density lipoprotein receptor-related protein 1 | Q07954 | -0.11 | 0.37 | -1.25 |
| 57 | Creatine kinase B-type | P12277 | -0.74 | 0.02 | -1.24 |
| 58 | Sarcoplasmic/endoplasmic reticulum calcium ATPase 2 | P16615 | -0.12 | 0.38 | -1.24 |
| 59 | Protein NDRG2 | Q9UN36 | -0.54 | 0.09 | -1.24 |
| 60 | ADP-ribosylation factor-like protein 3 | P36405 | -0.33 | 0.17 | -1.23 |
| 61 | Puromycin-sensitive aminopeptidase | P55786 | -0.14 | 0.36 | -1.23 |
| 62 | Tubulin beta-4A chain | P04350 | -0.30 | 0.18 | -1.22 |
| 63 | Serum albumin | P02768 | -0.17 | 0.29 | -1.22 |
| 64 | Glyceraldehyde-3-phosphate dehydrogenase | P04406 | -0.46 | 0.15 | -1.22 |
| 65 | Heterogeneous nuclear ribonucleoprotein U | Q00839 | -0.42 | 0.10 | -1.21 |
| 66 | Coronin-1A | P31146 | -0.18 | 0.31 | -1.21 |
| 67 | Tubulin beta chain | P07437 | -0.29 | 0.19 | -1.21 |
| 68 | Tubulin beta-4B chain | P68371 | -0.28 | 0.19 | -1.21 |
| 69 | Protein disulfide-isomerase A6 | Q15084 | -0.22 | 0.24 | -1.20 |
| 70 | Sodium/potassium-transporting ATPase subunit beta-2 | P14415 | -0.19 | 0.28 | -1.20 |

*Table S7: Ingenuity Pathway Analysis of diseases and biomarkers for top 38 differentially expressed proteins in myopic samples: Number of Molecules > 6.*

| **Categories** | **Diseases or Functions Annotation** | **p-value** | **Activation z-score** | **Molecules** | **# Molecules** |
| --- | --- | --- | --- | --- | --- |
| Cancer, Organismal Injury and Abnormalities | Extra pancreatic malignant tumor | 0.027 | 0.73 | ACOT7,ACTN4,AP2A1,ARF3,ARR3,ATP6V1B2,CDC37,CRYAA/LOC102724652,CRYAB,CRYBA1,CRYBA2,CRYBB2,CRYBB3,FGG,FMOD,GPX3,GSN,GSTM3,GULP1,HINT1,KCTD12,LUM,MYOM1,OGN,OPN1MW (includes others),PCBP2,PEBP1,PGD,PGK1,PSAT1,RDH10,RPL10A,RPS5,SPTBN1,VCL | 35 |
| Cancer, Organismal Injury and Abnormalities | Carcinoma | 0.024 |  | ACOT7,ACTN4,AP2A1,ARF3,ARR3,ATP6V1B2,CDC37,CRYAA/LOC102724652,CRYAB,CRYBA1,CRYBA2,CRYBB2,CRYBB3,FGG,FMOD,GPX3,GSN,GSTM3,GULP1,HINT1,KCTD12,LUM,MYOM1,OGN,OPN1MW (includes others),PCBP2,PEBP1,PGD,PGK1,PSAT1,RDH10,RPL10A,RPS5,SPTBN1,VCL | 35 |
| Cancer, Organismal Injury and Abnormalities | Abdominal cancer | 0.020 |  | ACOT7,ACTN4,AP2A1,ARF3,ARR3,ATP6V1B2,CDC37,CRYAA/LOC102724652,CRYAB,CRYBA1,CRYBA2,CRYBB2,CRYBB3,FGG,FMOD,GPX3,GSN,GSTM3,GULP1,HINT1,KCTD12,LUM,MYOM1,OGN,PCBP2,PEBP1,PGD,PGK1,PSAT1,RDH10,RPL10A,RPS5,SPTBN1,VCL | 34 |
| Cancer, Organismal Injury and Abnormalities | Head and neck tumor | 0.022 |  | ACOT7,ACTN4,AP2A1,ARF3,ARR3,ATP6V1B2,CDC37,CRYAA/LOC102724652,CRYAB,CRYBA1,CRYBB2,CRYBB3,FGG,FMOD,GPX3,GSN,GSTM3,GULP1,KCTD12,LUM,MYOM1,OGN,OPN1MW (includes others),PCBP2,PEBP1,PGD,PGK1,PSAT1,RDH10,RPS5,SPTBN1,VCL | 32 |
| Organismal Injury and Abnormalities | Soft tissue lesion | 0.012 |  | ACOT7,ACTN4,AP2A1,ARF3,ARR3,ATP6V1B2,CDC37,CRYAA/LOC102724652,CRYAB,CRYBA1,FGG,GSN,GSTM3,GULP1,KCTD12,LUM,MYOM1,OGN,OPN1MW (includes others),PCBP2,PGK1,RDH10,RPS5,SPTBN1,VCL | 25 |
| Neurological Disease, Organismal Injury and Abnormalities | Brain lesion | 0.026 |  | ACOT7,ACTN4,AP2A1,ARF3,ATP6V1B2,CDC37,CRYAA/LOC102724652,CRYAB,CRYBA1,FGG,GSN,GSTM3,GULP1,LUM,MYOM1,OGN,OPN1MW (includes others),PCBP2,PGK1,RDH10,RPS5,SPTBN1,VCL | 23 |
| Cell Death and Survival, Organismal Injury and Abnormalities | Necrosis | 0.000 | 0.88 | ACTN4,ARR3,ATP6V1B2,CDC37,CRYAA/LOC102724652,CRYAB,FMOD,GPX3,GSN,GULP1,HINT1,LUM,OGN,OPN1MW (includes others),PCBP2,PEBP1,PGD,PSAT1,RDH10,RPS5,SEPTIN2,SPTBN1 | 22 |
| Cancer, Neurological Disease, Organismal Injury and Abnormalities | Brain astrocytoma | 0.006 |  | ACOT7,ACTN4,AP2A1,ATP6V1B2,CRYAA/LOC102724652,CRYBA1,FGG,GSN,GSTM3,GULP1,LUM,MYOM1,OGN,OPN1MW (includes others),PCBP2,PGK1,RDH10,RPS5,SPTBN1,VCL | 20 |
| Cancer, Neurological Disease, Organismal Injury and Abnormalities | Grade 4 high grade glioma | 0.006 |  | ACOT7,ACTN4,AP2A1,ATP6V1B2,CRYAA/LOC102724652,CRYBA1,FGG,GSN,GSTM3,GULP1,LUM,MYOM1,OGN,OPN1MW (includes others),PCBP2,PGK1,RDH10,RPS5,SPTBN1,VCL | 20 |
| Cancer, Neurological Disease, Organismal Injury and Abnormalities | Grade 4 astrocytoma | 0.006 |  | ACOT7,ACTN4,AP2A1,ATP6V1B2,CRYAA/LOC102724652,CRYBA1,FGG,GSN,GSTM3,GULP1,LUM,MYOM1,OGN,OPN1MW (includes others),PCBP2,PGK1,RDH10,RPS5,SPTBN1,VCL | 20 |
| Cell Death and Survival | Apoptosis | 0.000 | 0.67 | ACTN4,ARR3,CDC37,CRYAA/LOC102724652,CRYAB,CRYBA1,CRYBB2,FMOD,GPX3,GSN,HINT1,LUM,OGN,PCBP2,PEBP1,PSAT1,SEPTIN2,SPTBN1,VCL | 19 |
| Organismal Injury and Abnormalities, Organismal Survival | Organismal death | 0.001 | -1.70 | ACTN4,CRYAB,FGG,GPX3,GSN,KCTD12,LUM,MYOM1,PCBP2,PGK1,PSAT1,RDH10,RPL10A,SEPTIN7,SPTBN1,VCL | 16 |
| Cancer, Organismal Injury and Abnormalities | Connective or soft tissue tumor | 0.001 |  | ARR3,ATP6V1B2,CRYAB,FMOD,GSTM3,HINT1,KCTD12,LUM,OGN,PCBP2,PGK1,SPTBN1,VCL | 13 |
| Neurological Disease, Organismal Injury and Abnormalities | Movement Disorders | 0.000 |  | ARR3,ATP6V1B2,CRYAB,FGG,FMOD,HINT1,LUM,PEBP1,PGK1,PSAT1,SEPTIN11,SPTBN1 | 12 |
| Gene Expression | Expression of RNA | 0.022 | 2.16 | ACTN4,CRYAA/LOC102724652,CRYAB,GSN,HINT1,LUM,MYOM1,PCBP2,PEBP1,RPL10A,RPS5 | 11 |
| Cell Death and Survival, Organismal Injury and Abnormalities | Cell death of tumor cell lines | 0.006 | 0.03 | ACTN4,CDC37,CRYAB,GSN,GULP1,HINT1,PCBP2,PEBP1,PGD,PSAT1,SEPTIN2 | 11 |
| Organismal Development | Abnormal morphology of body cavity | 0.001 |  | ACTN4,CRYAB,FMOD,GPX3,GSN,LUM,MYOM1,PGK1,RDH10,SPTBN1,VCL | 11 |
| Organismal Injury and Abnormalities | Non-malignant disorder | 0.008 |  | ATP6V1B2,CRYAB,FMOD,GPX3,GSTM3,HINT1,LUM,OGN,PSAT1,RDH10,SPTBN1 | 11 |
| Cellular Assembly and Organization | Assembly of organelle | 0.000 | 1.95 | ARF3,CRYAA/LOC102724652,CRYAB,FMOD,GSN,LUM,MYOM1,OGN,SEPTIN2,SEPTIN7 | 10 |
| Cellular Assembly and Organization, Cellular Function and Maintenance | Microtubule dynamics | 0.003 | 1.34 | ACTN4,AP2A1,CRYAA/LOC102724652,CRYAB,GSN,SEPTIN11,SEPTIN2,SEPTIN7,SPTBN1,VCL | 10 |
| Cardiovascular Disease, Cardiovascular System Development and Function, Organismal Injury and Abnormalities | Abnormal morphology of cardiovascular system | 0.000 |  | ACTN4,CRYAB,FMOD,GPX3,GSN,LUM,MYOM1,PGK1,SPTBN1,VCL | 10 |
| Organismal Development, Organismal Injury and Abnormalities | Abnormal morphology of thoracic cavity | 0.000 |  | CRYAB,FMOD,GPX3,GSN,LUM,MYOM1,PGK1,RDH10,SPTBN1,VCL | 10 |
| Organismal Development | Morphology of head | 0.000 |  | ATP6V1B2,CRYAA/LOC102724652,CRYBB2,FMOD,LUM,OGN,OPN1MW (includes others),RDH10,SPTBN1,VCL | 10 |
| Embryonic Development, Organismal Development, Tissue Development | Development of head | 0.000 |  | CRYAA/LOC102724652,CRYAB,CRYBA1,CRYBB2,FMOD,LUM,OGN,OPN1MW (includes others),RDH10,SPTBN1 | 10 |
| Cancer, Organismal Injury and Abnormalities, Renal and Urological Disease | Bladder cancer | 0.013 |  | ATP6V1B2,CDC37,CRYBA1,GPX3,GSN,KCTD12,MYOM1,PGK1,SPTBN1,VCL | 10 |
| Cellular Movement | Invasion of cells | 0.008 | 1.65 | ACTN4,CRYAB,FMOD,GSN,PEBP1,PSAT1,SEPTIN11,SEPTIN2,VCL | 9 |
| Cell Death and Survival, Organismal Injury and Abnormalities | Apoptosis of tumor cell lines | 0.012 | -0.33 | ACTN4,CDC37,CRYAB,GSN,HINT1,PCBP2,PEBP1,PSAT1,SEPTIN2 | 9 |
| Embryonic Development, Nervous System Development and Function, Organ Development, Organismal Development, Tissue Development, Visual System Development and Function | Formation of eye | 0.000 |  | CRYAA/LOC102724652,CRYAB,CRYBA1,CRYBB2,FMOD,LUM,OGN,OPN1MW (includes others),RDH10 | 9 |
| Hereditary Disorder, Ophthalmic Disease, Organismal Injury and Abnormalities | Hereditary Eye Disease | 0.000 |  | ARR3,CRYAA/LOC102724652,CRYAB,CRYBA1,CRYBA2,CRYBB2,CRYBB3,GSN,OPN1MW (includes others) | 9 |
| Cardiovascular Disease, Cardiovascular System Development and Function, Organ Morphology, Organismal Development, Organismal Injury and Abnormalities | Abnormal morphology of heart | 0.000 |  | CRYAB,FMOD,GPX3,GSN,LUM,MYOM1,PGK1,SPTBN1,VCL | 9 |
| Neurological Disease, Organismal Injury and Abnormalities, Psychological Disorders | Disorder of basal ganglia | 0.000 |  | ARR3,ATP6V1B2,CRYAB,FGG,HINT1,PEBP1,PGK1,PSAT1,SPTBN1 | 9 |
| Organismal Development, Organismal Injury and Abnormalities | Abnormal morphology of head | 0.001 |  | ATP6V1B2,CRYAA/LOC102724652,CRYBB2,FMOD,LUM,OGN,RDH10,SPTBN1,VCL | 9 |
| Neurological Disease, Organismal Injury and Abnormalities, Skeletal and Muscular Disorders | Neuromuscular disease | 0.002 |  | ARR3,ATP6V1B2,CRYAB,FGG,HINT1,PEBP1,PGK1,PSAT1,SPTBN1 | 9 |
| Cancer, Organismal Injury and Abnormalities, Renal and Urological Disease | Bladder carcinoma | 0.024 |  | ATP6V1B2,CDC37,CRYBA1,GPX3,GSN,KCTD12,MYOM1,PGK1,SPTBN1 | 9 |
| Developmental Disorder, Hereditary Disorder, Organismal Injury and Abnormalities | Familial congenital malformation | 0.025 |  | ATP6V1B2,CRYAA/LOC102724652,CRYAB,CRYBA1,CRYBA2,CRYBB3,GSN,PSAT1,RDH10 | 9 |
| Hereditary Disorder, Neurological Disease, Organismal Injury and Abnormalities | Familial encephalopathy | 0.029 |  | ARR3,ATP6V1B2,CRYAB,FGG,HINT1,PGK1,PSAT1,SEPTIN11,SPTBN1 | 9 |
| Cell Morphology, Cellular Assembly and Organization, Cellular Function and Maintenance | Formation of cellular protrusions | 0.009 | 2.21 | ACTN4,AP2A1,GSN,SEPTIN11,SEPTIN2,SEPTIN7,SPTBN1,VCL | 8 |
| Hereditary Disorder, Neurological Disease, Organismal Injury and Abnormalities, Psychological Disorders, Skeletal and Muscular Disorders | Huntington Disease | 0.000 |  | ARR3,ATP6V1B2,CRYAB,FGG,HINT1,PGK1,PSAT1,SPTBN1 | 8 |
| Cancer, Organismal Injury and Abnormalities | Benign solid tumor | 0.003 |  | ATP6V1B2,CRYAB,FMOD,HINT1,LUM,OGN,PSAT1,SPTBN1 | 8 |
| Cellular Function and Maintenance | Engulfment of cells | 0.000 | 1.76 | ACTN4,AP2A1,ATP6V1B2,CRYBA1,GSN,GULP1,LUM | 7 |
| Cellular Function and Maintenance | Endocytosis | 0.001 | 1.77 | ACTN4,AP2A1,ARR3,ATP6V1B2,GSN,GULP1,LUM | 7 |
| Cardiovascular System Development and Function, Cell Morphology, Organ Morphology, Organismal Development | Morphology of heart cells | 0.000 |  | CRYAB,FMOD,GPX3,LUM,MYOM1,SPTBN1,VCL | 7 |
| Embryonic Development, Nervous System Development and Function, Organ Development, Organ Morphology, Organismal Development, Tissue Development, Visual System Development and Function | Morphology of eye | 0.000 |  | CRYAA/LOC102724652,CRYBB2,FMOD,LUM,OGN,OPN1MW (includes others),RDH10 | 7 |
| Cellular Assembly and Organization, Tissue Development | Formation of filaments | 0.000 |  | CRYAA/LOC102724652,CRYAB,FMOD,GSN,LUM,MYOM1,OGN | 7 |
| Cardiovascular Disease, Cardiovascular System Development and Function, Organ Morphology, Organismal Development, Organismal Injury and Abnormalities | Enlargement of heart | 0.001 |  | CRYAB,GPX3,GSN,MYOM1,PGK1,SPTBN1,VCL | 7 |
| Organismal Injury and Abnormalities, Reproductive System Disease | Benign pelvic disease | 0.005 |  | FMOD,GPX3,GSTM3,HINT1,LUM,OGN,SPTBN1 | 7 |
| Organismal Injury and Abnormalities, Reproductive System Disease | Disorder of pregnancy | 0.007 |  | FGG,FMOD,GSN,GSTM3,OGN,PSAT1,VCL | 7 |
| Cellular Function and Maintenance | Endocytosis by eukaryotic cells | 0.000 | 1.73 | ACTN4,AP2A1,ATP6V1B2,GSN,GULP1,LUM | 6 |
| Cell Death and Survival, Organismal Injury and Abnormalities | Necrosis of epithelial tissue | 0.005 | 1.93 | CDC37,CRYAA/LOC102724652,CRYAB,GSN,LUM,SPTBN1 | 6 |
| Cell Death and Survival, Organismal Injury and Abnormalities | Cell death of connective tissue cells | 0.003 | 0.45 | ARR3,CRYAB,GSN,HINT1,LUM,OGN | 6 |
| Hereditary Disorder, Ophthalmic Disease, Organismal Injury and Abnormalities | Autosomal dominant cataract disease | 0.000 |  | CRYAA/LOC102724652,CRYAB,CRYBA1,CRYBA2,CRYBB2,CRYBB3 | 6 |
| Developmental Disorder, Ophthalmic Disease, Organismal Injury and Abnormalities | Congenital cataract | 0.000 |  | CRYAA/LOC102724652,CRYAB,CRYBA1,CRYBA2,CRYBB2,CRYBB3 | 6 |
| Embryonic Development, Nervous System Development and Function, Ophthalmic Disease, Organ Development, Organ Morphology, Organismal Development, Organismal Injury and Abnormalities, Tissue Development, Visual System Development and Function | Abnormal morphology of eye | 0.000 |  | CRYAA/LOC102724652,CRYBB2,FMOD,LUM,OGN,RDH10 | 6 |
| Embryonic Development, Organ Development, Organismal Development, Skeletal and Muscular System Development and Function, Tissue Development | Formation of muscle | 0.000 |  | ACTN4,CRYAB,FMOD,GSN,MYOM1,VCL | 6 |
| Cancer, Organismal Injury and Abnormalities | Subcutaneous tumor | 0.001 |  | ACTN4,FMOD,GPX3,KCTD12,LUM,OGN | 6 |
| Cancer, Organismal Injury and Abnormalities | Adenoma | 0.001 |  | CRYAB,FMOD,HINT1,LUM,PSAT1,SPTBN1 | 6 |
| Organismal Injury and Abnormalities, Psychological Disorders | Severe psychological disorder | 0.011 |  | ACOT7,ATP6V1B2,CRYAB,GSN,HINT1,MYOM1 | 6 |
| Hereditary Disorder, Organismal Injury and Abnormalities, Skeletal and Muscular Disorders | Hereditary myopathy | 0.013 |  | CRYAB,GSN,HINT1,LUM,MYOM1,VCL | 6 |
| Organismal Survival | Survival of organism | 0.023 |  | ACTN4,CRYAB,GSN,KCTD12,RDH10,VCL | 6 |

*Table S8. Ingenuity Pathway Analysis: Top Canonical Pathways for top 38 differentially expressed RPE exosomal proteins in myopic samples.*

| **Name** | **p-value** | **Overlap** |
| --- | --- | --- |
| Integrin Signaling | 3.59E-04 | 1.9 % 4/212 |
| RHOA Signaling | 1.03E-03 | 2.4 % 3/124 |
| Germ Cell-Sertoli Cell Junction Signaling | 2.53E-03 | 1.8 % 3/170 |
| Phototransduction Pathway | 3.39E-03 | 3.7 % 2/54 |
| Sertoli Cell-Sertoli Cell Junction Signaling | 4.34E-03 | 1.5 % 3/206 |

*Table S9. Potential RPE exosomal protein biomarkers for myopia based on STRING database, published data* (1,2)*, and current study.*

| **SN** | **Gene Symbol** | **Description** | **Current study** |
| --- | --- | --- | --- |
| 1 | ALDH3A2 | Aldehyde dehydrogenase 3 family member A2 |  |
| 2 | AP1B1 | Adaptor related protein complex 1 subunit beta 1 |  |
| 3 | APOE* | Apolipoprotein E |  |
| 4 | ARCN1 | Archain 1 |  |
| 5 | ARR3 | Arrestin 3 | Top 10 downregulated |
| 6 | ATP6V1A | ATPase H+ transporting V1 subunit A | Downregulated |
| 7 | ATP6V1B2 | ATPase H+ transporting V1 subunit B2 | Top 10 downregulated |
| 8 | CLU | Clusterin |  |
| 9 | COL12A1* | Collagen type XII alpha 1 chain | Unique |
| 10 | CRYAB | Crystallin alpha B | Downregulated |
| 11 | CRYBB1 | Crystallin beta B1 |  |
| 12 | CRYBB2 | Crystallin beta B2 | Top 10 downregulated |
| 13 | CRYGC | Crystallin gamma C |  |
| 14 | GNAT2 | G protein subunit alpha transducin 2 |  |
| 15 | GNB3 | G protein subunit beta 3 |  |
| 16 | HNRNPK | Heterogeneous nuclear ribonucleoprotein K |  |
| 17 | LRAT | Lecithin retinol acyltransferase |  |
| 18 | MAP2K1 | Mitogen-activated protein kinase kinase 1 |  |
| 19 | MYOC | Myocilin | Unique |
| 20 | PDE6C | Phosphodiesterase 6C |  |
| 21 | PEBP1 | Phosphatidylethanolamine binding protein 1 | Downregulated |
| 22 | PURA | Purine rich element binding protein A |  |
| 23 | RDH5 | Retinol dehydrogenase 5 |  |
| 24 | RHOA | Ras homolog family member A |  |
| 25 | RPE65 | Retinoid isomerohydrolase RPE65 |  |
| 26 | TGFBI | Transforming growth factor beta induced | Unique |
| 27 | SAG | S-antigen visual arrestin 1 | Unique |
| 28 | UCHL1 | Ubiquitin C-terminal hydrolase L1 | Top 10 downregulated |
| *Reported other family member protein | | | |

References:

1. Klingeborn M, Dismuke WM, Skiba NP, Kelly U, Stamer WD, Bowes Rickman C. Directional exosome proteomes reflect polarity-specific functions in retinal pigmented epithelium monolayers. Sci Rep. 2017 Jul 7;7(1):4901.

2. Szklarczyk D, Kirsch R, Koutrouli M, Nastou K, Mehryary F, Hachilif R, et al. The STRING database in 2023: protein-protein association networks and functional enrichment analyses for any sequenced genome of interest. Nucleic Acids Res. 2023 Jan 6;51(D1):D638–46.
